# A systematic review of neurocognition and social cognition in body dysmorphic disorder

**DOI:** 10.1177/00048674241309747

**Published:** 2025-01-07

**Authors:** Holmes à Court Katrina, Amy Malcolm, Wei Lin Toh, Susan L Rossell

**Affiliations:** 1 Centre for Mental Health, Swinburne University of Technology, Melbourne, VIC, Australia; 2 Psychiatry, St Vincent’s Hospital, Melbourne, VIC, Australia

**Keywords:** Body dysmorphic disorder (BDD), cognitive psychology, neurocognition, visual perception, selective attention, social cognition, executive function

## Abstract

**Objective:**

Neurocognitive underpinnings are implicated in the aetiology and maintenance of body dysmorphic disorder (BDD); however, inconsistent findings across a range of neurocognitive domains suggest that a comprehensive synthesis of the literature using a hierarchical framework of neurocognition is needed.

**Methods:**

A final search across OVID Medline, PsycNET, Scopus and Web of Science databases was conducted on 20 June 2024 to identify research that examined performance on behavioural tasks of objective neurocognition in BDD. Risk of bias was assessed using the Newcastle-Ottawa Scale. Fifty-four studies aligned with the following inclusion criteria: (1) full-text; (2) peer-reviewed; (3) published in English; (4) employed a neurocognitive task with an objective outcome and (5) involved a case-controlled paradigm consisting of BDD and healthy control samples. Findings were synthesised according to neurocognitive sub-domains viewed as a hierarchy from basic to higher-level domains.

**Results:**

Neurocognitive differences in BDD relative to controls were identified at almost all levels of the hierarchy, most consistently in the upper domains of executive function and social cognition. Vulnerabilities were also demonstrated in the sub-domains of visual perception of faces, Gestalt processing, selective attention to faces and verbal memory. Methodological limitations or the influence of neurocognitive sub-groups may contribute to inconsistencies across the literature.

**Conclusions:**

Although neurocognitive differences appear central to BDD, a picture of neurocognitive heterogeneity emerged with the salience of stimuli important and a likely bias to local-over-global processing demonstrated across the domains.

Body dysmorphic disorder (BDD) is a mental disorder characterised by excessive preoccupation with perceived flaws in appearance accompanied by repetitive behaviours and/or mental acts causing clinically significant distress and/or dysfunction (American Psychiatric Association, 2013). While its aetiology remains unclear, BDD presents clinically as an enduring perception of the self as deformed or grotesque, which negatively impacts all facets of life (Rossell, 2023). The prominence of misperception and fixed beliefs implicates neurocognitive aspects in the development and maintenance of symptoms.

Research has indicated significantly reduced performance in BDD compared to healthy controls (HCs) across multiple neurocognitive domains. These include visual organisation (Kerwin et al., 2014), attention (Toh et al., 2015a), memory (Deckersbach et al., 2000) and planning/problem solving (Dunai et al., 2010). The largest empirical study of neurocognition in BDD (*n* = 65) reported significant impairments in inhibition/flexibility, speed of processing, working memory, visual and verbal learning and reasoning/problem-solving (Malcolm et al., 2021). Another comprehensive study found a global cognition deficit in BDD using the Repeatable Battery for the Assessment of Neuropsychological Status (RBANS) with a very large effect (Toh et al., 2015a; BDD < HC *d* = 1.36). However, findings of neurocognitive differences in BDD have not been consistently ascertained across the literature (e.g. Greenberg et al., 2018; Rossell et al., 2014).

Johnson et al. (2018) performed the first systematic review and meta-analysis of neurocognitive functioning in BDD, indicating significantly reduced performance relative to HCs across 23 studies, with small to medium pooled effect sizes across the domains of interpretative biases (*g* = 0.30), memory (*g* = 0.56) and selective attention (*g* = 0.60), although no significant group differences were documented for local visual processing (*g* = 0.35). However, the review did not explore a broad range of neurocognitive functions beyond the four selected domains of interest.

This systematic review sought to address this gap, as well as incorporating additional studies published since the work by Johnson et al. (2018). Specifically, we aimed to examine a comprehensive range of neurocognitive investigations performed in BDD as viewed through the perspective of a hierarchically organised framework of neurocognition (Harvey, 2019). As shown in Figure 1, this framework posits that cognition starts with basic sensory and perceptual lower-order processes and builds in complexity towards higher-order operations, such as those involving language and executive functions (Harvey, 2019). Social cognition can also be considered as an advanced neurocognitive process reliant on executive functions, and thus has been included as an additional domain at the top of the hierarchy. The hierarchy model presents neurocognitive functions as inter-connected domains that increase in complexity and can influence each other through a feedback loop. Thus, basic processes both influence and are controlled by higher-order domains. Examination of patterns of neurocognitive differences in BDD according to this hierarchy can help clarify inconsistent findings to date by contextualising potential impairments in one function against those in connected domains. Furthermore, given the importance of social cognition to the cognitive-behavioural theoretical basis, as well as the lived experience of BDD, domains involving emotion processing and theory of mind, which used validated tasks with correct/incorrect outcomes, were included.

**Figure 1.**
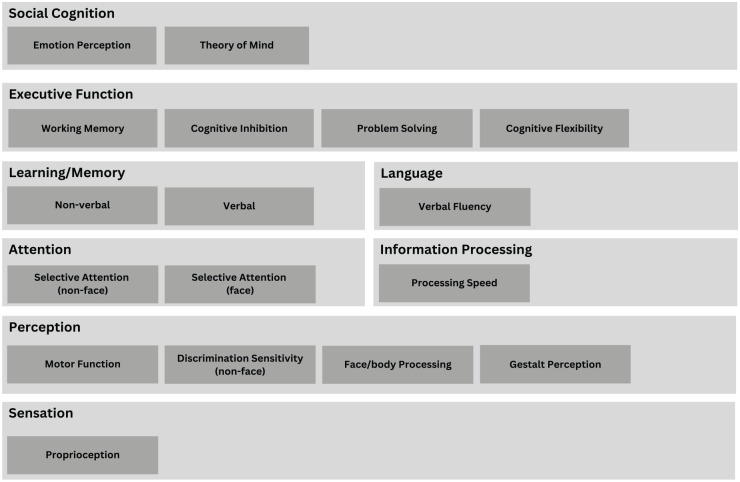
Hierarchy of neurocognitive domains.

## Method

### Search and selection

The systematic review protocol was developed in accordance with the Preferred Reporting Items for Systematic Reviews and Meta-Analyses protocol (PRISMA; Moher et al., 2009) and was registered with the international prospective register of systematic reviews, PROSPERO (ID: CRD42020191074). The search strategy was amended since registration to increase the number of databases included, using PsycNET, Web of Science, Scopus and OVID Medline. The final search was conducted on June 20, 2024. Search terms were chosen to be inclusive of body dysmorphic disorder (e.g. ‘BDD’, ‘Dysmorphophobia’, ‘Body dysmorphia’) and cognition (e.g. ‘neurocognition’), and the search syntax was tailored for each database. Search strategy, including full syntax and search strings for each database, are reported in Supplemental Materials. Inclusion criteria were (1) full-text; (2) peer-reviewed; (3) published in the English language; (4) employed a neurocognitive task with an objective outcome (e.g. accuracy or response time) and (5) involved a case-controlled paradigm consisting of BDD and HC samples. A two-staged screening process was used to determine eligibility of records for inclusion. At stage one, KhàC and CC independently screened the titles, abstracts, and keywords of all records. Where eligibility was unclear, articles were retained for the second stage of the screening process. At stage two, KhàC, CC and AM independently screened the full text of retained articles for eligibility. Discrepancies that arose at stage two were resolved by author consensus. Inter-rater reliability was 100%.

### Effect sizes

To support interpretation of findings, Cohen’s *d* values for group differences between BDD and HC participants were calculated, where possible. Where data on group means and standard deviations were not available, Cohen’s *d* values were calculated from either a t-statistic, F-value, or Pearson’s r value using an effect size conversion programme (Sullivan and Feinn, 2012).

### Risk of bias

Risk of bias was assessed by two independent reviewers (KHàC and SLR) using the Newcastle-Ottawa Scale to examine case selection (method of case/HC definition, origin of samples; maximum 4 points), comparability of cases and controls (study design comparability, e.g. differences in age, sex, education; maximum 2 points) and exposure (exposure method, comparability of exposure, non-response; maximum 3 points; Wells et al., 2011). Studies were rated 0–9, with 0–2 denoting poor quality, 3–5 fair quality and 6–9 high quality (see Supplemental Material, Table 4).

### Hierarchical framework

The reporting of findings according to cognitive domains was organised according to the bottom-up hierarchical framework reflecting increasing levels of complexity involved in each cognitive operation (Harvey, 2019). While there is overlap between domains, and generally tasks tap into multiple domains, tasks were allocated according to their predominant domain. To reveal subtle differences in contributions to cognitive processing, tasks that examined multiple variables were further split, and the variables were allocated to the appropriate sub-domain. For example, some studies using the Rey-Osterrieth Complex Figure Task (RCFT) reported both organisation, which was allocated to the perception domain, and delayed recall, which was allocated to the memory domain (e.g. Deckersbach et al., 2000). To help synthesise the extensive results, summaries for each cognitive domain are provided.

## Results

### Search

**Figure 2.**
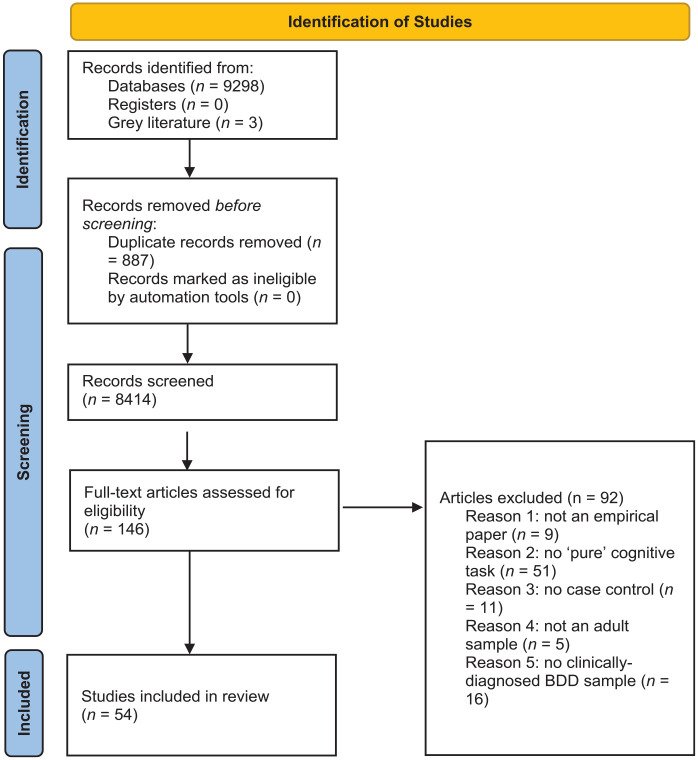
PRISMA flow diagram showing record screening and selection.

The search provided 8,441 records after the removal of duplicates, from which 54 articles were identified for inclusion (see Figure 2).

### Risk of bias

Table 1 demonstrates that overall risk of bias within studies was low. The minimum quality of studies retained was fair quality (one study), with the vast majority being of high quality (53 studies; for a detailed analysis, see Supplemental Material, Table 4).

**Table 1.** Quality analysis: Newcastle-Ottawa Scale (NOS) for case-controlled studies.

Study	Selection	Comparability	Exposure	Score
Buhlmann et al. (2006)	⋆⋆⋆⋆	⋆	⋆⋆⋆	8
Buhlmann et al. (2011a)	⋆⋆⋆⋆	⋆⋆	⋆⋆⋆	9
Buhlmann et al. (2004)	⋆⋆⋆⋆	⋆⋆	⋆⋆⋆	9
Buhlmann et al. (2002)	⋆⋆⋆⋆	⋆	⋆⋆⋆	8
Buhlmann et al. (2014)	⋆⋆⋆⋆	⋆	⋆⋆⋆	8
Buhlmann et al. (2011b)	⋆⋆⋆⋆	⋆	⋆⋆⋆	8
Buhlmann et al. (2015)	⋆⋆⋆⋆	⋆⋆	⋆⋆⋆	9
Buhlmann et al. (2013)	⋆⋆⋆⋆	⋆⋆	⋆⋆⋆	9
Chen et al. (2023)	⋆⋆⋆	⋆⋆	⋆⋆⋆	8
Deckersbach et al. (2000)	⋆⋆⋆⋆	⋆⋆	⋆⋆⋆	9
Dunai et al. (2010)	⋆⋆⋆⋆	⋆⋆	⋆⋆⋆	9
Fang et al. (2020)	⋆⋆⋆⋆	⋆	⋆⋆⋆	8
Feusner et al. (2010a)	⋆⋆⋆⋆	⋆⋆	⋆⋆	8
Feusner et al. (2011a)	⋆⋆⋆⋆	⋆⋆	⋆⋆	8
Feusner et al. (2010b)	⋆⋆⋆⋆	⋆⋆	⋆⋆	8
Feusner et al. (2007)	⋆⋆⋆⋆	⋆⋆	⋆⋆	8
Giannopoulos et al. (2022)	⋆⋆⋆⋆	⋆	⋆⋆	7
Grace et al. (2019)	⋆⋆⋆⋆	⋆⋆	⋆⋆	8
Greenberg et al. (2014)	⋆⋆⋆⋆	⋆⋆	⋆⋆	8
Greenberg et al. (2018)	⋆⋆⋆⋆	⋆	⋆⋆	7
Grocholewski et al. (2012)	⋆⋆⋆⋆	⋆	⋆⋆⋆	8
Hanes (1998)	⋆⋆		⋆	3
Hartmann et al. (2015)	⋆⋆⋆⋆	⋆⋆	⋆⋆	8
Hübner et al. (2016)	⋆⋆⋆⋆	⋆⋆	⋆⋆	8
Jefferies et al. (2012)	⋆⋆⋆⋆	⋆⋆	⋆⋆	8
Jefferies-Sewell et al. (2017)	⋆⋆	⋆⋆	⋆⋆⋆	7
Kaplan et al. (2014)	⋆⋆⋆⋆	⋆⋆	⋆⋆	8
Kerwin et al. (2014)	⋆⋆⋆⋆	⋆⋆	⋆⋆	8
Kollei et al. (2017)	⋆⋆⋆⋆	⋆⋆	⋆	7
Lambrou et al. (2011)	⋆⋆⋆⋆	⋆⋆	⋆⋆⋆	9
Li et al. (2015)	⋆⋆⋆⋆	⋆⋆	⋆⋆	8
Malcolm et al. (2021)	⋆⋆⋆⋆	⋆	⋆⋆	7
Monzani et al. (2013)	⋆⋆⋆⋆	⋆⋆	⋆⋆	8
Moody et al. (2015)	⋆⋆⋆⋆	⋆⋆	⋆⋆⋆	9
Onken et al. (2024)	⋆⋆⋆⋆	⋆⋆	⋆⋆⋆	9
Pikoos et al. (2024)	⋆⋆⋆⋆	⋆⋆	⋆⋆⋆	9
Reese et al. (2010)	⋆⋆⋆⋆	⋆	⋆⋆⋆	8
Reese et al. (2011a)	⋆⋆⋆⋆	⋆⋆	⋆⋆⋆	9
Reese et al. (2011b)	⋆⋆⋆⋆	⋆⋆	⋆⋆⋆	9
Ritter et al. (2020)	⋆⋆⋆⋆	⋆⋆	⋆⋆	8
Rossell et al. (2014)	⋆⋆⋆⋆	⋆⋆	⋆⋆	8
Silverstein et al. (2015)	⋆⋆⋆⋆	⋆	⋆⋆	7
Stangier et al. (2008)	⋆⋆⋆⋆	⋆⋆	⋆⋆⋆	9
Toh et al. (2015a)	⋆⋆⋆⋆	⋆⋆	⋆⋆⋆	9
Toh et al. (2015b)	⋆⋆⋆⋆	⋆⋆	⋆⋆⋆	9
Toh et al. (2017a)	⋆⋆⋆⋆	⋆⋆	⋆⋆⋆	9
Toh et al. (2017b)	⋆⋆⋆⋆	⋆⋆	⋆⋆⋆	9
Toh et al. (2017c)	⋆⋆⋆⋆	⋆⋆	⋆⋆⋆	9
Waldorf et al. (2019)	⋆⋆⋆⋆	⋆⋆	⋆⋆⋆	9
Wang et al. (2024)	⋆⋆⋆⋆	⋆	⋆⋆⋆	8
Wilhelm et al. (2003)	⋆⋆⋆⋆	⋆⋆	⋆⋆⋆	9
Wong et al. (2022)	⋆⋆⋆⋆	⋆⋆	⋆⋆⋆	9
Yaryura-Tobias et al. (2002)	⋆⋆⋆		⋆⋆⋆	6
Yousefi et al. (2020)	⋆⋆⋆	⋆⋆	⋆⋆⋆	8

The designator ⋆ denotes that study has met criteria outlined in accordance with Newcastle-Ottawa Quality Assessment Scale for case-control studies.

### Study characteristics

Table 2 shows the characteristics of the retained studies.

**Table 2.** Characteristics of included studies (*N* = 54).

Characteristic	Count
Total participants	2089
Total participants with BDD	1033
Total HC participants	1060
Average number of total participants	45
Average number of participants with BDD	23
Range of sample sizes (total)	20–135
Range of sample sizes (BDD)	9–65
Percentage of BDD samples who are female	67%
Number of studies employing only female participants	3
Number of studies employing only male participants	1
Mean Age BDD (*SD*)	31 years (8.94)
Mean Age HC (*SD*)	30 years (9.20)
Cognitive measures employed	31
Cognitive domains examined	7
Cognitive sub-domains examined	17

Age range is not included as it was not widely reported across the studies.

### Cognitive findings

Table 3 presents a summary of the cognitive findings across the studies.

**Table 3.** Summary of included studies, tasks employed and key behavioural findings by cognitive domain.

Domain	Study	Sample	Task/Variable	Group outcome	Effect size (Cohen’s d)
**Sensation**					**0.34–0.52**
	Kaplan et al. (2014)	17 BDD, 17 HC	RHI: Synchronous	BDD = HC	0.34
			RHI: Asynchronous	BDD = HC	0.52
**Perception**					**0.03–0.85**
Motor function	Hanes (1998)	14 BDD, 24 HC	Purdue Pegboard (dexterity)	BDD = HC	0.13
Visual perception					0.03–0.85
	Buhlmann et al. (2014)	35 BDD, 35 HC	Shape modification detection accuracy	BDD = HC	0.03
Non-face discrimination	Feusner et al. (2011)	14 BDD, 14 HC	Object-matching: Accuracy	BDD = HC	0.14
			Object-matching: RT	BDD < HC	0.85
	Feusner et al. (2007)	12 BDD, 13 HC	Oval matching: Accuracy	BDD = HC	+
			Oval matching: RT	BDD = HC	+
	Li et al. (2015)	20 BDD, 20 HC	Object-matching: Accuracy	BDD = HC	+
			Object-matching: RT	BDD = HC	+
	Moody et al. (2015)	20 BDD, 20 HC	Oval matching: Accuracy	BDD = HC	LSF: + HSF: +
			Oval matching: RT	BDD = HC	LSF: + HSF: +
	Reese et al. (2010)	20 BDD, 20 HC	Dot-symmetry: Accuracy	BDD = HC	0.40
			Dot-symmetry: RT	BDD = HC	0.05
	Rossell et al. (2014)	14 BDD, 14 HC	Contour integration task	BDD = HC	0.29
			VOSP % Correct: Incomplete letters	BDD = HC	0.14
			VOSP % Correct: Silhouettes	BDD = HC	0.31
			VOSP % Correct: Object decision	BDD = HC	0.68
			VOSP % Correct: Progress silhouettes	BDD = HC	0.45
Face discrimination	Buhlmann et al. (2004)	20 BDD, 20 HC	Benton faces: Accuracy	BDD = HC	0.15
	Fang et al. (2020)	20 BDD, 28 HC	QSFID: Accuracy	BDD = HC	0.71
	Feusner et al. (2010a)	12 BDD, 11 HC	IR: Accuracy (emotional faces)	BDD < HC	+
			IR: Accuracy (neutral faces)	BDD = HC	+
			IR: RT (emotional faces)	BDD < HC	0.54
			IR: RT (neutral faces)	BDD < HC	0.22
	Feusner et al. (2010b)	18 BDD, 17 HC	Faces vs Morph: Accuracy (short)	BDD = HC	+
			Faces vs Morph: Accuracy (long)	BDD = HC	+
	Feusner et al. (2007)	12 BDD, 13 HC	Neutral faces vs Ovals: Accuracy	BDD = HC	+
			Neutral faces vs Ovals: RT	BDD = HC	+
	Jefferies et al. (2012)	12 BDD, 16 HC	Famous Faces: Recognition accuracy	BDD = HC	0.48
	Li et al. (2015)	20 BDD, 20 HC	Neutral faces vs Ovals: Accuracy	BDD = HC	+
			Neutral faces vs Ovals: RT	BDD = HC	+
	Monzani et al. (2013)	25 BDD, 25 HC	CT: Face top/bottom matching: Accuracy	BDD = HC	+
			CT: Face top/bottom matching: RT	BDD = HC	+
	Ritter et al. (2020)	16 BDD, 16 HC	Discrimination own face: Accuracy	BDD = HC	0.55
			Discrimination other face: Accuracy	BDD = HC	0.04
			Discrimination own face: RT	BDD = HC	0.74
			Discrimination other face: RT	BDD = HC	0.50
	Toh (2017c)	21 BDD, 21 HC	Own face matching: Accuracy	BDD < HC	1.19
			Own face matching: RT	BDD > HC	0.50
Modification detection	Buhlmann et al. (2014)	35 BDD, 35 HC	Other face discrimination accuracy	BDD = HC	0.36
			Other face non-existent manipulations	BDD > HC	0.72
	Hübner et al. (2016)	32 BDD, 32 HC	Other face discrimination accuracy	BDD = HC	0.13
			Other face modification discrepancy	BDD = HC	0.24
	Lambrou et al. (2011)	50 BDD, 50 HC	Change detection accuracy (own face)	BDD > HC	0.90
			Change detection accuracy (other face)	BDD = HC	0.07
	Onken et al. (2024)	25 BDD, 25 HC	Facial symmetry: Accuracy (other face)	BDD = HC	0.21
	Pikoos et al. (2024)	30 BDD, 27 HC	Featural modification: Accuracy (same)	BDD = HC	0.41
			Featural modification: Accuracy (different)	BDD = HC	0.22
			Featural modification: RT (same)	BDD = HC	0.26
			Featural modification: RT (same)	BDD = HC	0.27
	Reese et al. (2010)	20 BDD, 20 HC	Facial symmetry: Accuracy (other face)	BDD = HC	0.11
			Facial symmetry: RT (other face)	BDD = HC	0.07
	Stangier et al. (2008)	21 BDD, 20 HC	Facial manipulation accuracy (other face)	BDD > HC	1.00
			Change rating accuracy (other face)	BDD > HC	1.32
	Yaryura-Tobias et al. (2002)	10 BDD, 10 HC	Own face non-existent distortion	BDD > HC	BDD = 50%, HC = 0%
Gestalt Non-face Visual Organisation	Deckersbach et al. (2000)	17 BDD, 17 HC	RCFT: Figure copy organization	BDD < HC	0.83
			RCFT: Immediate recall organization	BDD = HC	0.48
	Giannopoulos et al. (2022)	18 BDD, 18 HC	Ebbinghaus illusion accuracy	BDD = HC	+
			Vertical-horizontal illusion accuracy	BDD = HC	
			Zöllner illusion accuracy	BDD = HC	
			Poggendorff illusion accuracy	BDD = HC	
			Kanizsa’s triangle illusion accuracy	BDD = HC	
			Café wall illusion accuracy	BDD = HC	
			Hering illusion accuracy	BDD = HC	
			Shepard tables illusion accuracy	BDD = HC	
			Sander illusion accuracy	BDD = HC	
	Greenberg et al. (2018)	20 BDD, 20 HC	RCFT: Figure copy organisation	BDD < HC	0.97
			RCFT: Immediate recall organisation	BDD = HC	0.48
			RCFT: Delayed recall organisation	BDD < HC	0.86
	Silverstein et al. (2015)	20 BDD, 20 HC	Ebbinghaus illusion accuracy Contour integration task	BDD = HC BDD = HC	0.74
	Yaryura-Tobias et al. (2002)	10 BDD, 10 HC	WAIS-III: Perceptual organisation factor	BDD = HC	+
			WAIS-III: Picture completion	BDD = HC	+
			WAIS-III: Block design	BDD = HC	+
			WAIS-III: Matrix reasoning	BDD = HC	+
	Yousefi et al. (2020)	19 BDD, 26 HC	RCFT: Copy organisation	BDD = HC	+
Gestalt Non-face Configural Processing	Kerwin et al. (2014)	18 BDD, 17 HC	Embedded Figures Task (EFT): Accuracy	BDD < HC	0.84
			NT: Local trial accuracy	BDD = HC	0.58
			NT: Global trial accuracy	BDD < HC	0.94
			NT: RT (local)	BDD < HC	1.25
			NT: RT (global)	BDD < HC	1.00
			NT: RT (switch condition)	BDD < HC	0.98
			NT: RT (non-switch condition)	BDD < HC	1.26
			NT: Set condition (global-local)	BDD < HC	1.37
			NT: Set condition (local-global)	BDD < HC	1.00
			NT: Set condition (local-local)	BDD < HC	1.09
			NT: Set condition (global-global)	BDD < HC	0.97
	Monzani et al. (2013)	25 BDD, 25 HC	NT: RT	BDD = HC	0.04
	Pikoos et al. (2024)	30 BDD, 27 HC	NT: Accuracy	BDD = HC	0.29
			NT: RT	BDD = HC	0.21
Gestalt Distortion Processing	Feusner et al. (2011)	14 BDD, 14 HC	Object matching: Accuracy	BDD = HC	LSF: 0.52, HSF: 0.10
			Object matching: RT	BDD < HC	LSF: 0.89, HSF: 0.64
	Feusner et al. (2007)	12 BDD, 13 HC	Oval matching: Accuracy	BDD = HC	+
			Oval matching: RT	BDD = HC	+
	Li et al. (2015)	20 BDD, 20 HC	Object matching: Accuracy	BDD = HC	LSF: + HSF: +
			Object matching: RT	BDD = HC	LSF: + HSF: +
	Moody et al. (2015)	20 BDD, 20 HC	Face matching: Accuracy	BDD = HC	LSF: + HSF: +
			Face matching: RT	BDD = HC	LSF: + HSF: +
	Pikoos et al. (2024)	30 BDD, 27 HC	Face matching: RT (same)	BDD = HC	0.26
			Face matching: RT (different)	BDD = HC	0.28
			Face matching: Accuracy (same)	BDD = HC	0.20
			Face matching: Accuracy (different)	BDD = HC	0.28
	Wang et al. (2024)	9 BDD, 10 HC	Face matching: Accuracy	BDD = HC	LSF: 0.84
Gestalt Inversion Processing	Feusner et al. (2010b)	18 BDD, 17 HC	Face inversion: Own face recognition: Accuracy	BDD = HC(short)BDD = HC(long)	+
			Face inversion: RT	BDD = HC(short)BDD < HC(long)	+
	Jefferies et al. (2012)	12 BDD, 16 HC	Inverted face recognition (FFT) accuracy	BDD > HC	1.82
	Monzani et al. (2013)	25 BDD, 25 HC	Inversion effects matching: Accuracy	BDD = HC	0.45
			Inversion effects face/house matching: RT	BDD = HC	0.42
	Pikoos et al. (2024)	30 BDD, 27 HC	Accuracy inversion effect: PFA	BDD > HC	
			Other-inverted faces: Accuracy	BDD = HC	0.02
			Other-inverted faces: RT (same)	BDD > HC	0.70
			Other-inverted faces: RT (different)	BDD > HC	0.62
	Ritter et al. (2020)	16 BDD, 16 HC	Own-inverted faces: Accuracy	BDD = HC	0.29
			Other-inverted faces: Accuracy	BDD = HC	0.41
			Own-inverted faces: RT	BDD = HC	0.44
			Other-inverted faces: RT	BDD = HC	0.57
	Toh et al. (2017c)	21 BDD, 21 HC	Accuracy inversion effect: Mooney faces	BDD < HC	0.60
			Accuracy inversion effect: Objects	BDD < HC	0.83
			Object inversion: Accuracy	BDD > HC	0.81
			Object inversion: RT	BDD > HC	0.81
**Attention**					**0.12–1.5**
Selective (non-face) attention	Malcolm et al. (2021)	65 BDD, 70 HC	MATRICS: Attention/vigilance	BDD = HC	0.37
	Toh et al. (2015a)	21 BDD, 21 HC	RBANS: Attention Index	BDD < HC	1.50
			RBANS: Digit Span Forward	BDD < HC	1.27
			RBANS: Coding	BDD = HC	0.82
	Toh et al. (2017a)	21 BDD, 21 HC	Emotional stroop: Eye-tracking	BDD = HC	Medium/large
	Yousefi et al. (2020)	19 BDD, 26 HC	WMS-III: Digit Span Forward	BDD = HC	0.25
			WMS-III: Digit Span Backward	BDD < HC	1.35
Selective face/body attention	Greenberg et al. (2014)	19 BDD, 20 HC	Negative mean bias score (unattractive)	BDD > HC	+
			Fixation (negative bias)	BDD = HC	0.29
			Dwell (negative bias)	BDD < HC	0.20
	Grocholewski et al. (2012)	20 BDD, 20 HC	Own face: Fixations	BDD > HC	0.74
			Other face: Fixations	BDD > HC	0.52
			Own face: Fixation durations	BDD = HC	0.43
			Other face: Fixation durations	BDD = HC	0.26
	Kollei et al. (2017)	19 BDD, 21 HC	Own attractive features: Dwell time	BDD < HC	0.75
			Own unattractive features: Dwell time	BDD > HC	0.91
			Own attractive features: Fixation count	BDD = HC	0.83
			Own unattractive features: Fixation count	BDD > HC	0.68
	Toh (2017c)	21 BDD, 21 HC	Own face: Fixations	BDD < HC	1.18
			Feature Fixation Index	BDD < HC	1.35
			Feature Duration Index	BDD < HC	1.62
			Other face: Fixations	BDD < HC	1.31
			Feature Fixation Index	BDD < HC	1.00
			Feature Duration Index	BDD < HC	1.00
	Waldorf et al. (2019)	24 BDD, 24 HC	Eye-tracking own body: Attractive area	BDD > HC	+
			Eye-tracking own body: Unattractive area	BDD < HC	+
	Wong (2022)	37 BDD, 30 HC	Eye-tracking own face: Fixations	BDD < HC	+
**Processing speed**					**0.00–0.85**
	Jefferies-Sewell et al. (2017)	12 BDD, 16 HC	Stop Signal Task: RT	BDD = HC	0.00
	Malcolm et al. (2021)	65 BDD, 70 HC	MATRICS: Speed of Processing Index	BDD < HC	0.85
	Toh et al. (2015a)	21 BDD, 21 HC	RBANS: Coding	BDD = HC	0.82
**Language**					**0.05 – 1.14**
	Rossell et al. (2014)	14 BDD, 14 HC	COWA: Semantic fluency (foods)	BDD < HC	1.14
			COWA: Semantic fluency (body)	BDD < HC	1.09
			COWA: Semantic fluency (animals)	BDD = HC	0.91
			COWA: Phonological fluency (letter F)	BDD = HC	0.25
			COWA: Phonological fluency (letter A)	BDD = HC	0.20
			COWA: Phonological fluency (letter S)	BDD = HC	0.05
**Learning and memory**					**0.03 – 2.33**
Non-verbal memory	Deckersbach et al. (2000)	17 BDD, 17 HC	RCFT: Copy accuracy	BDD = HC	0.21
			RCFT: Immediate recall	BDD < HC	0.90
	Dunai et al. (2010)	14 BDD, 14 HC	CANTAB: Pattern recognition	BDD = HC	0.03
	Fang et al. (2020)	20 BDD, 28 HC	Cambridge Face Memory Test (CFMT)	BDD = HC	0.63
	Greenberg et al. (2018)	20 BDD, 20 HC	RCFT: Copy accuracy	BDD = HC	0.13
			RCFT: Immediate recall	BDD = HC	0.30
			RCFT: Delayed recall	BDD = HC	0.36
	Hanes (1998)	14 BDD, 24 HC	RCFT: Copy accuracy	BDD = HC	0.14
			RCFT: Delayed recall accuracy	BDD = HC	0.10
	Malcolm et al. (2021)	65 BDD, 70 HC	MATRICS: Visual learning	BDD < HC	1.02
	Toh et al. (2015a)	21 BDD, 21 HC	RBANS: Visuospatial Construction Index	BDD = HC	0.40
	Yousefi et al. (2020)	19 BDD, 26 HC	RCFT: Copy accuracy	BDD < HC	1.13
			RCFT: Immediate recall	BDD < HC	1.39
	Wang et al. (2024)	9 BDD, 10 HC	Face recognition learning	BDD = HC	0.89
Verbal learning and memory	Deckersbach et al. (2000)	17 BDD, 17 HC	CVLT: Recall (1–5 trials)	BDD < HC	0.98
			CVLT: Short-delay recall	BDD < HC	0.91
			CVLT: Long-delay recall	BDD < HC	0.79
			CVLT: Mean % recall	BDD = HC	0.11
			CVLT: Recognition discriminability	BDD < HC	0.70
			CVLT: Semantic clustering (organisation)	BDD < HC	0.95
			CVLT: Serial clustering	BDD = HC	0.30
	Hanes (1998)	14 BDD, 24 HC	RAVLT: Recall (1–5 trials)	BDD = HC	0.11
			RAVLT: Delayed recall	BDD = HC	0.13
	Malcolm et al. (2021)	65 BDD, 70 HC	MATRICS: Verbal learning	BDD < HC	0.51
	Reese et al. (2011b)	20 BDD, 20 HC	Word recognition (128 word)	BDD = HC	+
	Toh et al. (2015a)	21 BDD, 21 HC	RBANS: Immediate Memory Index	BDD < HC	1.21
			RBANS: Delayed Memory Index	BDD = HC	0.58
			RBANS: Language Index	BDD = HC	0.40
	Yousefi et al. (2020)	19 BDD, 26 HC	WMS-III VL: Immediate recall	BDD < HC	1.33
			WMS-III VL: Delay recall	BDD < HC	1.32
			WMS-III VL: Thematic 1	BDD < HC	2.33
			WMS-III VL: Thematic 2	BDD < HC	1.83
			VPA: Immediate recall	BDD = HC	0.20
			VPA: Delayed recall	BDD < HC	0.72
**Executive function**					**0.05–3.24**
Working memory	Chen et al. (2023)	26 BDD, 28 HC	CANTAB: SWM (accuracy)	BDD = HC	0.39
			CANTAB: SWM (strategy)	BDD = HC	0.80
	Dunai et al. (2010)	14 BDD, 14 HC	CANTAB: SWM	BDD < HC	1.23
			CANTAB: Spatial span	BDD = HC	0.80
	Malcolm et al. (2021)	65 BDD, 70 HC	MATRICS: Working Memory Index	BDD < HC	1.16
	Yousefi et al. (2020)	19 BDD, 26 HC	WMS-III: Digit Span Total	BDD < HC	0.94
			WMS-III: Spatial span	BDD < HC	0.83
Cognitive inhibition	Chen et al. (2023)	26 BDD, 28 HC	Stop Signal Task: RT	BDD = HC	0.22
	Hanes (1998)	14 BDD, 24 HC	Stroop test	BDD > HC	0.66
	Jefferies-Sewell et al. (2017)	12 BDD, 16 HC	CGT: Proportion of bets	BDD < HC	3.24
			CGT: Delay aversion	BDD > HC	0.94
			CGT: Risk taking	BDD < HC	1.25
			CGT: Quality of decision-making	BDD = HC	0.27
			CGT: Deliberation time	BDD = HC	0.55
			Stop Signal Task: RT	BDD > HC	0.87
Cognitive flexibility	Chen et al. (2023)	26 BDD, 28 HC	CANTAB: IED: Accuracy	BDD = HC	0.05
	Greenberg et al. (2018)	20 BDD, 20 HC	CANTAB: IED: Accuracy	BDD < HC	0.66
	Jefferies-Sewell et al. (2017)	12 BDD, 16 HC	CANTAB: IED: Stages completed	BDD < HC	1.21
			CANTAB: IED: Total errors	BDD < HC	1.47
			CANTAB: IED: Shift errors	BDD < HC	1.32
	Kerwin et al. (2014)	18 BDD, 17 HC	Navon Task: Switch condition	BDD < HC	+
	Malcolm et al. (2021)	65 BDD, 70 HC	MATRICS: Inhibition/flexibility scale	BDD < HC	0.49
Emotional inhibition	Buhlmann et al. (2002)	16 BDD, 16 HC	Emotional stroop: BDD threat words	BDD > HC	0.82
			Stroop: BDD positive	BDD > HC	1.03
			Stroop: General threat	BDD = HC	0.52
			Stroop: General positive	BDD = HC	0.48
			Stroop: Neutral	BDD = HC	0.20
			Stroop: Current concern hypothesis	BDD > HC	1.19
			Stroop: Emotionality hypothesis	BDD > HC	0.95
	Buhlmann et al. (2011b)	36 BDD, 36 HC	AGN: Attractiveness beliefs	BDD > HC	0.70
	Hartmann et al. (2015)	23 BDD, 22 HC	AGN: Accuracy	BDD = HC	+
			BAAS	BDD > HC	+
	Jefferies-Sewell et al. (2017)	12 BDD, 16 HC	AGN: Accuracy	BDD < HC	0.90
			AGN: RT (positive words)	BDD < HC	1.81
			AGN: RT (negative words)	BDD = HC	+
			AGN: Commission errors (CE)	BDD > HC	0.96
			AGN: CE (positive words)	BDD > HC	1.34
			AGN: CE (negative words)	BDD > HC	0.79
			AGN: CE (neutral words)	BDD = HC	0.29
			AGN: Omission errors (OE)	BDD > HC	2.00
			AGN: OE (positive words)	BDD > HC	1.09
			AGN: OE (negative words)	BDD > HC	1.34
			AGN: OE (neutral words)	BDD = HC	0.20
	Malcolm et al. (2021)	65 BDD, 70 HC	MSC: Managing emotions	BDD = HC	0.22
	Rossell et al. (2014)	14 BDD, 14 HC	Emotional stroop: X-Sequence (accuracy)	BDD = HC	0.12
			Emotional Stroop: X-Sequence (RT)	BDD = HC	0.31
	Toh et al. (2017a)	21 BDD, 21 HC	Emotional Stroop: RT (negative words)	BDD < HC	0.62
	Wilhelm et al. (2003)	14 BDD, 14 HC	NP: Threatening words (short trials)	BDD = HC	0.10
			NP: Threatening words (long trials)	BDD = HC	0.28
			NP: Non-threat words (short trials)	BDD = HC	0.52
			NP: Non-threat words (long trials)	BDD = HC	0.78
Planning and Problem-solving	Dunai et al. (2010)	14 BDD, 14 HC	Stockings of Cambridge (SOC): Solved	BDD < HC	1.15
			SOC: Perfect solutions	BDD < HC	1.27
			SOC: Excess moves	BDD > HC	1.52
			SOC: Speed	BDD = HC	+
	Hanes (1998)	14 BDD, 24 HC	NToL: Problems solved	BDD < HC	0.89
	Malcolm et al. (2021)	65 BDD, 70 HC	MATRICS: Reasoning/problem solving	BDD < HC	.80
	Reese et al. (2011b)	20 BDD, 20 HC	BRT: Draws to decision (neutral)	BDD = HC	+
			BRT: Draws change score (difficulty)	BDD = HC	+
			BRT: Draws to decision (confidence)	BDD = HC	+
			BRT: Draws to decision (self-referent)	BDD = HC	+
	Yaryura-Tobias et al. (2002)	10 BDD, 10 HC	Wisconsin Card Sorting Test	BDD = HC	+
**Social cognition**					**0.03 – 4.15**
Emotion perception	Buhlmann et al. (2004)	20 BDD, 20 HC	EFT: Emotion recognition accuracy	BDD < HC	0.95
			Accuracy: Neutral	BDD < HC	1.14
			Accuracy: Disgust	BDD < HC	1.12
			Misinterpretation bias (disgust)	BDD > HC	1.12
			Misinterpretation bias (anger)	BDD > HC	1.12
	Buhlmann et al. (2006)	18 BDD, 18 HC	EFT: Overall (self) recognition accuracy	BDD < HC	0.97
			Accuracy: Neutral	BDD < HC	1.12
			Misinterpretation bias (anger)	BDD > HC	1.18
			Misinterpretation bias (disgust)	BDD > HC	0.71
			Overall (other) recognition accuracy	BDD = HC	0.64
	Buhlmann et al. (2011a)	34 BDD, 34 HC	EFT: Emotion recognition accuracy	BDD = HC	0.08
			Accuracy: Neutral	BDD < HC	0.69
	Fang et al. (2020)	20 BDD, 28 HC	MEI: Overall accuracy	BDD = HC	0.11
			QSFD: Overall recognition accuracy	BDD = HC	0.60
	Grace et al. (2019)	19 BDD, 21 HC	PFA: Overall recognition accuracy	BDD < HC	0.93
			Accuracy: Angry	BDD < HC	0.33
			Accuracy: Sad	BDD < HC	0.79
			Accuracy: Neutral	BDD < HC	0.95
			Response time: Angry	BDD > HC	0.29
			Response time: Happy	BDD > HC	0.51
			Response time: Neutral	BDD > HC	0.01
	Jefferies et al. (2012)	12 BDD, 16 HC	FEEST: Emotion recognition accuracy	BDD = HC	0.87
			Accuracy: Fear	BDD < HC	1.16
	Pikoos et al. (2024)	30 BDD, 27 BDD	PFA: Emotion recognition accuracy	BDD = HC	0.21
			PFA: Emotion recognition RT	BDD = HC	0.02
	Rossell et al. (2014)	14 BDD, 14 HC	PFA: Emotion recognition accuracy	BDD = HC	+
	Toh et al. (2015b)	21 BDD, 21 HC	PFA: Emotion recognition accuracy	BDD < HC	0.91
			Misinterpretation bias: Anger	BDD > HC	+
			Accuracy: Anger	BDD < HC	0.72
			Accuracy: Fear	BDD < HC	0.35
			Accuracy: Sad	BDD < HC	0.61
	Toh et al. (2017c)	21 BDD, 21 HC	PFA: Emotion recognition accuracy	BDD < HC	0.23
			PFA: Emotion recognition RT	BDD = HC	0.16
			Own face: Recognition accuracy	BDD < HC	0.96
			Own face: RT	BDD = HC	0.50
Theory of mind	Buhlmann et al. (2013)	31 BDD, 31 HC	RMET: Accuracy	BDD = HC	0.21
			RMET: Accuracy (positive emotions)	BDD = HC	0.34
			RMET: Accuracy (negative emotions)	BDD = HC	0.03
			RMET: Accuracy (neutral emotions)	BDD = HC	0.15
	Buhlmann et al. (2015)	35 BDD, 35 HC	MASC: SIA (thoughts/intentions)	BDD < HC	0.82
			MASC: SIA (emotions)	BDD = HC	0.14
	Fang et al. (2020)	20 BDD, 28 HC	RMET: Accuracy	BDD > HC	4.15

The designator + denotes no effect size calculated. AGN, Affective Go/No-Go Task; BRT, Beads Reasoning Task; BAAS, Beliefs about Appearance Scale; BDD, Body Dysmorphic Disorder; CGT, Cambridge Gambling Test; CANTAB, Cambridge Neuropsychological Test Automated Battery; CVLT, California Verbal Learning Test; CT, Composite Task; COWA, Controlled Oral Word Association; EFT, Ekman and Friesen Task; FEEST, Facial Expression of Emotions Stimulus and Test; HC, Healthy Controls; HSF, High spatial frequency; IR, Identity Recognition; IED, Intra-Extra Dimensional Set-shifting Task; LSF, Low Spatial Frequency; MSC, Mayer-Salovey-Caruso; MATRICS, MATRICS Consensus Cognitive Battery; MEI, Morphed Emotion Identification; MASC, Movie for the Assessment of Social Cognition; NT, Navon Task; NP, Negative Priming; NToL, New Tower of London; NSF, Normal spatial frequency; PFA, Pictures of Facial Affect; QSFID, Queen Square Face Discrimination Test; RMET, Reading the Mind in the Eyes Task; RT, response time; RAVLT, Rey Auditory Verbal Learning Task; RCFT, Rey Complex Figure Task; RBANS, Repeatable Battery for the Assessment of Neuropsychological Status; RHI, Rubber Hand Illusion; SIA, Social Inference Accuracy; SWM, Spatial Working Memory; SOC, Stockings Of Cambridge; WAIS-III, The Wechsler Adult Intelligence Scale-III; VL, verbal learning; VPA, Verbal Paired Association; VOSP, visual object and space perception; WMS-III, Wechsler Memory Scale-III; BDD-YBOCS, Yale-Brown Obsessive-Compulsive Scale modified for Body Dysmorphic Disorder.

### Sensation

A single study found no significant differences in proprioception using the rubber hand illusion across both synchronous and asynchronous stimulation conditions (Kaplan et al., 2014).

### Perception

#### Motor function

A single study found no significant difference in motor function in BDD using the Purdue Pegboard (Hanes, 1998).

#### Visual perception

To distinguish the perception of general stimuli from those which may be of increased pertinence to individuals with BDD, this domain was categorised into sub-categories of non-face discrimination, face discrimination, face modification detection and Gestalt perception. We further categorised the Gestalt studies into organisation, configural processing, distortion and inversion effects.

##### Non-face discrimination

Visual discrimination of non-face stimuli was examined in eight studies that predominantly measured object-matching (Buhlmann et al., 2014; Feusner et al., 2007, 2011; Lambrou et al., 2011; Li et al., 2015; Moody et al., 2015; Reese et al., 2010; Rossell et al., 2014). Across these, 15 variables showed no significant differences in BDD, with small to moderate effect sizes. Only a single variable showed a significantly slower response time with a large effect size; however, this study had a small sample (BDD; *N* = 14; Feusner et al., 2011). A further study showed a medium to large effect size for reduced accuracy in BDD, although this finding did not reach statistical significance (Rossell et al., 2014).

##### Face discrimination

Eleven studies examined face discrimination: the ability to discriminate between photos of different faces and objects. Four variables showed significant differences in BDD. Feusner et al. (2010a) identified reduced accuracy and slower response times discriminating emotional faces as well as slower response times for neutral faces. This pattern of findings was confirmed by Toh et al. (2017c) using neutral Pictures of Facial Affect (PFA) and own-face stimuli with moderate to large effect sizes. Furthermore, Toh et al. (2017c) demonstrated an aversion to focus on both own and other face-processing, regardless of preoccupation foci.

Sixteen variables, encompassing accuracy or response times, displayed no significant differences in face discrimination in BDD, with effect sizes ranging from small to large. These studies employed diverse measures including the Benton Facial Recognition Test (BFRT; Buhlmann et al., 2004), Queen Square Face Discrimination Test (QSFD; Fang et al., 2020), Composite Face Matching Task (Monzani et al., 2013), Famous Faces Task (FFT; Jefferies et al., 2012), Penn Faces stimuli (Feusner et al., 2007, 2010a, 2010b; Li et al., 2015) and own faces (Ritter et al., 2020). Possible ceiling effects may account for some of these findings.

##### Modification detection

Eight studies investigated facial modification detection (Buhlmann et al., 2014; Hübner et al., 2016; Lambrou et al., 2011; Onken et al., 2024; Pikoos et al., 2024; Reese et al., 2010; Stangier et al., 2008; Yaryura-Tobias et al., 2002). Four variables showed atypical modification processing. Lambrou et al. (2011) demonstrated significantly enhanced accuracy in modification detection to own faces with a large effect size, and Stangier et al. (2008) showed significantly greater other-face accuracy under timed conditions. Buhlmann et al. (2014) found significantly increased reporting of non-existent changes in other faces. Yaryura-Tobias et al. (2002) demonstrated significantly less-accurate facial modification detection, with half of their BDD sample (of whom 80% had predominantly facial foci of concern) incorrectly detecting modifications to own faces when none existed. No incorrect detections were reported among healthy controls. These findings have been argued to support the notion of atypical aesthetic sensitivity for faces in BDD (Buhlmann et al., 2014; Lambrou et al., 2011; Stangier et al., 2008; Yaryura-Tobias et al., 2002).

Eleven other variables, however, showed no significant differences in accuracy or response times for other face change detection (Buhlmann et al., 2014; Hübner et al., 2016; Lambrou et al., 2011; Pikoos et al., 2024) or other face symmetry preference (Onken et al., 2024; Reese et al., 2010). Variations in study design may account for the inconsistent findings. For example, computer-generated stimuli were employed in the study by Buhlmann et al. (2014), whereas others presented computer-modified naturalistic images (Hübner et al., 2016; Lambrou et al., 2011; Onken et al., 2024; Pikoos et al., 2024; Reese et al., 2010; Stangier et al., 2008; Yaryura-Tobias et al., 2002). Stangier et al. (2008) examined only women, presenting colour photos of a single woman. Whereas Yaryura-Tobias et al. (2002) employed mixed-gender stimuli but examined a predominantly male BDD sample, and Reese et al. (2010) employed black-and-white photos and allowed extended viewing time.

##### Gestalt non-face visual organisation

Gestalt non-face visual organisation was examined in six studies (Deckersbach et al., 2000; Giannopoulos et al., 2022; Greenberg et al., 2018; Silverstein et al., 2015; Yaryura-Tobias et al., 2002; Yousefi et al., 2020). Three variables revealed significantly diminished performance on the RCFT in terms of copy organisation and delayed recall accuracy, with large effect sizes (Deckersbach et al., 2000; Greenberg et al., 2018). Compared to controls, individuals with BDD demonstrated impaired search and recall strategies when asked to replicate the complex figure, focusing on smaller details at the expense of larger, important design features.

However, analysis of 15 other variables using a variety of tasks, including illusion tasks (Giannopoulos et al., 2022), a contour integration task (Silverstein et al., 2015), the RCFT (organisation; Yousefi et al., 2020) and the perceptual organisation components of the Wechsler Adult Intelligence Scale-III (WAIS-III: Perceptual; Yaryura-Tobias et al., 2002), detected no significant differences in perceptual organisation, although moderate effect sizes for immediate recall on the RCFT (organisation; Deckersbach et al., 2000; Greenberg et al., 2018) and Ebbinghaus illusion task (Silverstein et al., 2015) were found. Findings of preserved immediate recall but impaired copy organisation have been proposed to stem from atypical organisational strategies characteristic in BDD, relying predominantly on detail-oriented processing while de-prioritising global processing (Deckersbach et al., 2000).

##### Gestalt non-face configural

Gestalt (non-face) configural processing was investigated in three studies utilising the Navon Task (NT; Kerwin et al., 2014; Monzani et al., 2013; Pikoos et al., 2024) and Embedded Figures Task (EFT; Kerwin et al., 2014). There were significant differences with large effect sizes across eight variables (indicating reduced accuracy or response times) on the NT (Kerwin et al., 2014). In contrast, Pikoos et al. (2024) and Monzani et al. (2013) found no significant differences in NT accuracy, albeit utilising a different methodology with additional instructions to focus attention on global or local stimuli.

##### Gestalt distortion

Gestalt distortion processing was investigated in six studies employing high and low spatial frequency-altered photos of objects (Feusner et al., 2011; Li et al., 2015) and faces (Feusner et al., 2007; Li et al., 2015; Moody et al., 2015; Pikoos et al., 2024; Wang et al., 2024). BDD participants showed significantly slower response times on two variables for both low and high spatial frequency objects with moderate to large effect sizes (Feusner et al., 2011).

However, 17 variables showed no significant behavioural differences in accuracy or response time for objects, own/other faces (Feusner et al., 2007, 2011; Li et al., 2015; Moody et al., 2015; Pikoos et al., 2024; Wang et al., 2024).

##### Gestalt inversion

Gestalt inversion processing was examined in six studies utilising inverted face tasks (Feusner et al., 2010b; Monzani et al., 2013; Pikoos et al., 2024; Ritter et al., 2020), the FFT (Jefferies et al., 2012) and the Mooney Faces Task (Toh et al., 2017b). Six variables indicated significantly reduced face or object-inversion effects based on response times or accuracy. These include response time for long-duration stimuli measured by Feusner et al. (2010b) and significantly increased inverted face recognition accuracy with a large effect size reported by Jefferies et al. (2012). Toh et al. (2017b) identified significantly reduced face and object inversion effects, alongside more accurate and faster object identification, with medium to large effect sizes. Contrary findings were demonstrated by Pikoos et al. (2024), who found significantly larger face-inversion effects across both ‘same’ and ‘different’ trials using response time.

However, 12 further behavioural variables exhibited no significant differences. These include no significant accuracy differences for both short- and long-duration stimuli, as reported by Feusner et al. (2010b), as well as no difference in response time for short-duration stimuli. Similarly, Monzani et al. (2013) found no significant differences in both accuracy and response times, and Ritter et al. (2020) found no significant differences in short-duration stimuli accuracy or response time across both own and other faces, findings which were mirrored by accompanying neurobiological evidence.

#### Summary

The current literature findings examining visual perception employing behavioural tasks in BDD are mixed. Only limited evidence exists for impairments in discriminating non-face stimuli (Feusner et al., 2011), while there is a larger body of evidence supporting atypical face discrimination (Feusner et al., 2010a; Toh et al., 2017c) and considerably more indicating differences in facial modification detection (Buhlmann et al., 2014; Lambrou et al., 2011; Stangier et al., 2008; Yaryura-Tobias et al., 2002). In the gestalt perception literature, substantial evidence indicated atypical, non-face, configural (Kerwin et al., 2014), inverted face/object perception (Feusner et al., 2010b; Jefferies et al., 2012; Pikoos et al., 2024; Toh et al., 2017b) and a potential predominance of detail-oriented, local-over-global visual processing.

### Attention/processing speed

#### Selective (non-face) attention

Selective (non-face) attention was examined in four studies (Malcolm et al., 2021; Toh et al., 2015a, 2017a; Yousefi et al., 2020). Utilising the RBANS, Toh et al. (2015a) demonstrated significantly reduced attention using the Digit Span Forwards and the Attention Index with large effect sizes. This was confirmed by Yousefi et al. (2020) employing the Digit Span Backward of the Wechsler Memory Scale-III (WMS-III). Toh et al. (2017a) also showed disorganised eye-tracking on the Emotional Stroop Task (a word-reading task involving BDD-relevant stimuli), suggesting attention deficits in BDD.

Two further behavioural variables showed no significant differences in non-face attention on the Digit Span Forwards and WMS-III (Yousefi et al., 2020) and MATRICS (Malcolm et al., 2021).

#### Selective face/body attention

Selective attention to faces and bodies using eye-tracking technology was examined in six studies. Reduced attention was found across four variables (both dwell time and fixations) when viewing own (Toh et al., 2017c; Wong et al., 2022) and other faces (Toh et al., 2017c). Grocholewski et al. (2012) showed significantly increased fixations to own or other faces (especially to areas corresponding to a perceived defect), although there were no significant differences in dwell time. Waldorf et al. (2019) showed significantly increased attention to attractive own-body features and reduced attention to unattractive own-body features. Kollei et al. (2017) showed this same pattern in faces with large effect sizes for dwell time and fixations, except for fixations of own-face attractive features, which were not significantly different from controls. Greenberg et al. (2014) showed significantly increased fixations for own least-attractive facial features and significantly increased dwell time for least-attractive features in both own and other faces, with small effect sizes.

#### Processing speed

Processing speed was examined in three studies (Jefferies-Sewell et al., 2017; Malcolm et al., 2021; Toh et al., 2015a). The Speed of Processing Index of the MATRICS battery demonstrated significantly slower processing in a large sample with a large effect size (Malcolm et al., 2021), the coding sub-test of the RBANS found no significant differences but a large effect size (Toh et al., 2015a) and the Stop Signal Task (SST) demonstrated no significant differences (Jefferies-Sewell et al., 2017). Of the limited literature examining processing speed in BDD, the finding of Malcolm et al. (2021) using a large sample and demonstrating significantly slower processing with a large effect is the most pertinent of all in this sub-domain.

#### Summary

Selective attention to faces and bodies emerged as an important area of cognitive difference in BDD, with all studies demonstrating significant variations, some exhibiting large effect sizes.

### Language

A single study examined language (Rossell et al., 2014). Two out of three tests of semantic fluency showed significantly reduced verbal learning capabilities with large effect sizes, and three tests of phonological fluency showed no significant differences with only small effect sizes.

### Learning and memory

#### Non-verbal memory

Nine studies examined non-verbal memory and learning (Deckersbach et al., 2000; Dunai et al., 2010; Fang et al., 2020; Greenberg et al., 2018; Hanes, 1998; Malcolm et al., 2021; Toh et al., 2015a; Wang et al., 2024; Yousefi et al., 2020). Four variables showed significant differences in BDD. Two showed significantly poorer immediate recall on the RCFT – in which subjects were asked to copy and recall a complex figure drawing – with large effect sizes (Deckersbach et al., 2000; Yousefi et al., 2020). Yousefi et al. (2020) demonstrated significantly poorer RCFT copy accuracy, in which the BDD group recalled more detailed aspects of the figure at the expense of overall accuracy. In the largest sample, Malcolm et al. (2021) showed significantly poorer visual learning with a large effect size using the MATRICS.

Seven further variables showed no significant differences. Small to moderate effect sizes were found using the Cambridge Neuropsychological Test Automated Battery (CANTAB) pattern recognition (Dunai et al., 2010), Cambridge Face Memory Test (CFMT; Fang et al., 2020) and RCFT (Deckersbach et al., 2000; Greenberg et al., 2018; Hanes, 1998; Toh et al., 2015a). While Wang et al. (2024) found no significant difference in distorted face learning, they demonstrated a large effect size, and the accompanying neurobiological data showed differences in BDD. In some cases, ceiling effects and task demands were suggested as explanations for these non-significant findings (e.g. Dunai et al., 2010; Toh et al., 2015b). The intact RCFT copy accuracy but impaired organisation finding of Deckersbach et al. (2000) implicates organisational strategy as a possible mechanism for memory impairment in BDD.

#### Verbal learning and memory

Six studies examined verbal learning and memory (Deckersbach et al., 2000; Hanes, 1998; Malcolm et al., 2021; Reese et al., 2011b; Toh et al., 2015a; Yousefi et al., 2020). Six variables examining immediate recall showed large effect sizes for both short and long delays (Deckersbach et al., 2000; Yousefi et al., 2020). Two variables demonstrated significantly poorer verbal learning with moderate effect sizes using the MATRICS (Malcolm et al., 2021) and Verbal Paired Association (Yousefi et al., 2020). The Immediate Memory Index of the RBANS showed a significant deficit in story memory with a large effect size. Impaired story recall with a large effect size was also found (Toh et al., 2015a).

However, findings of significant differences in verbal learning and memory in BDD were not found consistently. The Language and Delayed Memory Indexes of the RBANS showed no significant differences, although all tests showed large effect sizes, and ceiling effects were observed in some sub-tests (Toh et al., 2015a). Three further tests found no significant differences in word recall (Hanes, 1998; Reese et al., 2011b). The preserved memory for lists, but impaired story memory, reported by Toh et al. (2015a) implies a specific impairment in gist understanding. This suggests that salient memory tasks, rather than simple recall, may be more difficult in BDD.

#### Summary

While there are inconsistencies in findings across the literature, taken together, it appears that individuals with BDD experience significant deficits across multiple domains of memory. Deficits in both non-verbal and verbal memory and learning are demonstrated in large samples and with large effect sizes. While some variables showed no significant deficits, patterns of impairments, notably that of immediate memory deficits but intact language learning, have been found repeatedly (Deckersbach et al., 2000; Hanes, 1998; Toh et al., 2015a). Furthermore, individuals with BDD demonstrated significant difficulties with immediate verbal recall (Deckersbach et al., 2000; Yousefi et al., 2020), as well as memory for gist meanings of complex linguistic material (Toh et al., 2015a).

### Executive function

#### Working memory

Working memory was examined in four studies (Chen et al., 2023; Dunai et al., 2010; Malcolm et al., 2021; Yousefi et al., 2020), revealing consistent findings across three. Four variables demonstrated significantly poorer working memory in BDD with large effect sizes for visual working memory (Dunai et al., 2010), verbal working memory (Malcolm et al., 2021) and visual and verbal working memory (Yousefi et al., 2020). The significant and large finding of Malcolm et al. (2021) obtained using a large sample is of particular note. Furthermore, Yousefi et al. (2020) found that individuals with BDD performed comparably to controls on the forward component of the Digit Span sub-test but exhibited significantly poorer performance on the backward component.

However, significant working memory differences in BDD were not observed in two studies. Despite a large effect size observed on the Spatial Span in the study by Dunai et al. (2010), no significant differences were noted in accuracy and strategy. In addition, Chen et al. (2023) did not find significant group differences for accuracy in their study using the CANTAB Spatial Working Memory.

#### Cognitive inhibition

Across the three studies examining cognitive inhibition (Chen et al., 2023; Hanes, 1998; Jefferies-Sewell et al., 2017), five variables using the Stroop Test (Hanes, 1998), Cambridge Gambling Test (CGT; proportion of bets, risk taking and delay aversion) and SST (response time; Jefferies-Sewell et al., 2017) showed significantly poorer performance in BDD with large effect sizes. While two further variables using the CGT (quality of decision-making and deliberation time) did not reach statistical significance, they demonstrated moderate to large effect sizes (Jefferies-Sewell et al., 2017). Two further variables measured through the SST (response time: Chen et al., 2023) and CGT (quality of decision-making; Jefferies-Sewell et al., 2017) found no significant differences demonstrating small effect sizes.

#### Cognitive flexibility

Cognitive flexibility was investigated in five studies with differences demonstrated across six variables (Greenberg et al., 2018; Jefferies-Sewell et al., 2017; Kerwin et al., 2014; Malcolm et al., 2021). This included significant differences on the CANTAB: Intra-Extra Dimensional Set-shifting Task (IED) with moderate to large effect sizes (Greenberg et al., 2018; Jefferies-Sewell et al., 2017), while Kerwin et al. (2014) reported significantly slower response times in the switch condition of the NT, and Malcolm et al. (2021) identified significantly poorer flexibility using the cognitive flexibility scale of the MATRICS.

#### Emotional inhibition

Findings in emotional inhibition yielded mixed results across eight studies. Toh et al. (2017a) found significantly slower response times for both BDD-negative and BDD-positive words on the Emotional Stroop task. Furthermore, eye-tracking data in the same study revealed disorganised viewing strategies in BDD, involving both avoidance and extended focus. Similarly, Buhlmann et al. (2002) found significant interference from BDD-positive words in the BDD group. Two further variable outcomes showed significantly reduced accuracy and response times for BDD-positive words on the Affective Go/No-Go Task (AGN; Jefferies-Sewell et al., 2017). In addition, utilising the Emotional Stroop, Buhlmann et al. (2011b) reported significantly greater beliefs in the importance of attractiveness among individuals with BDD with a moderate effect size.

In contrast, no significant differences in response times were identified for BDD-negative words (Buhlmann et al., 2002; Jefferies-Sewell et al., 2017), accuracy and response latency on a modified Stroop task using body-related words (Rossell et al., 2014), emotion management (Malcolm et al., 2021), AGN (Hartmann et al., 2015) and negative priming tasks comparing appearance-related threat and non-threat words (Wilhelm et al., 2003).

#### Planning and problem-solving

Planning and problem-solving was examined in five studies. Five variables consistently demonstrated significantly diminished planning and problem-solving ability in BDD using the Stockings of Cambridge (Dunai et al., 2010), the New Towers of London (Hanes, 1998) and the MATRICS (Malcolm et al., 2021), all with large effect sizes. These deficits manifested as both significantly slower and less-accurate problem-solving and poorer planning skills.

However, two variables, evaluated through the Beads Reasoning Task (Reese et al., 2011a) and the Wisconsin Card Sorting Test (Yaryura-Tobias et al., 2002), did not show significant differences in planning and problem-solving.

#### Summary

Impairments in executive function in BDD were most pronounced in the sub-domains of working memory, cognitive flexibility and cognitive inhibition. There was some, but not consistent, evidence of planning and problem-solving deficits. Findings in the emotional inhibition sub-domain were least consistent.

### Social cognition

#### Emotion perception

Emotion perception was investigated across 10 studies. Five variables indicated significantly reduced overall emotion perception in BDD, most with large effect sizes (Buhlmann et al., 2004, 2006; Grace et al., 2019; Toh et al., 2015b, 2017c). Two variables did not demonstrate general reduced accuracy but revealed significant differences in specific emotions (Buhlmann et al., 2011a; Jefferies et al., 2012). Jefferies et al. (2012) observed a large effect size for impairment in recognising fear, while Buhlmann et al. (2011a) reported a moderate effect size for the recognition of neutral faces. Jefferies et al. (2012) also noted a large effect for impairment in overall perception using the Facial Expression of Emotions Stimulus and Test, although this did not reach statistical significance. Similarly, Fang et al. (2020) found a moderate effect but no significant difference in overall emotion perception using the, QSFD and Rossell et al. (2014) found a trend towards an anger recognition bias. While Buhlmann et al. (2006) noted a significant, large effect for overall emotion recognition in a self-referent condition, the findings for the other-referent condition did not reach statistical significance, although they did note a medium effect. Buhlmann et al. (2011a) reported no significant differences using a seven-emotion recognition task. Similarly, Pikoos et al. (2024) found no significant differences in emotion recognition accuracy or response time. Task variation across the emotion perception literature is significant including the type and number of emotions presented, the presentation manner and timing and the colour and type of the images. These variations in task characteristics have been suggested as possible explanations for the inconsistent findings (Pikoos et al., 2024).

More consistent findings have revealed significantly reduced accuracy and slower response times for negative emotions such as anger, sadness, fear and disgust and for neutral expressions (Buhlmann et al., 2004, 2006, 2011a; Toh et al., 2015b).

#### Theory of mind

Theory of mind has been examined in three studies. Two variables showed differences, with Buhlmann et al. (2015) showing significantly reduced social inference (thoughts and intentions) accuracy with moderate to large effect sizes on the Movie for the Assessment of Social Cognition (MASC). Fang et al. (2020) also revealed a significant difference, demonstrating a large effect size, for reduced complex emotion recognition on the Reading the Mind in the Eyes Task (RMET). In contrast, Buhlmann et al. (2013) found no significant differences and only small effect sizes across three variables employing the RMET, and Buhlmann et al. (2015) showed no significant difference on the emotion recognition component of the MASC.

#### Summary

Extensive research attention has been dedicated to exploring emotion perception in BDD, and despite some inconsistencies, indications point towards likely deficits in emotion recognition for facial stimuli, and preliminarily for own faces in individuals with BDD. The limited scope of studies and variations in outcomes underscores the need for further research examining theory of mind.

**Figure 3.**
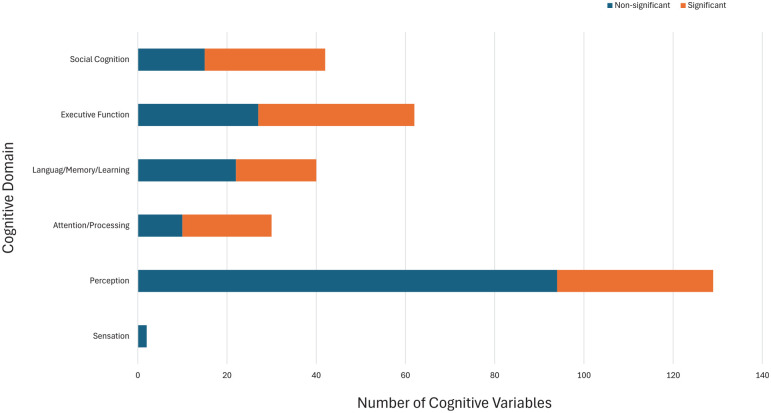
Figure demonstrating the significant differences in BDD according to the cognitive hierarchy. Cognitive variables *N* = 306.

Figure 3 summarises the results (as displayed comprehensively in Table 3) at a high level, showing the cognitive variables and domains examined across the studies.

## Discussion

This systematic review aimed to synthesise findings relevant to the behavioural measurement of cognition in BDD, using a cognitive hierarchy framework. A definitive cognitive profile for individuals with BDD was not uncovered; however, the findings evidenced differences between BDD and HC participants across all levels of the cognitive hierarchy which may be interrelated.

Atypical visual perception (Feusner et al., 2010b), especially of emotionally salient stimuli (Lambrou et al., 2011; Toh et al., 2017c), represents a likely cognitive marker in BDD. Moreover, evidence for local-over-global processing in BDD was found using varied tasks across all sub-domains of visual perception, as well as tasks involving selective attention to faces, non-verbal memory, emotion recognition and theory of mind. Thus, the influence of emotional salience and locally biased visual perception appears to be present across all levels of the cognition hierarchy. When neurological evidence was considered in addition to the behavioural findings, this processing tendency in BDD was corroborated (e.g. Feusner et al., 2007, 2010a, 2011; Li et al., 2015). The cognitive-behavioural model of BDD proposes that a cognitive style which preferences detail over holistic processing of visual information underlies BDD’s development and maintenance (Fang and Wilhelm, 2015). The findings of this review support this model; the most consistent behavioural findings are in relation to BDD global/local processing differences across the domains (i.e. in modification detection, Gestalt visual processing, selective attention to faces/bodies, learning and memory, working memory and emotion perception). However, the review findings further suggest that visual disturbances may be experienced heterogeneously across individuals and tasks. Cognitive sub-groups, such as individuals who are selectively versus broadly impaired, may underlie behavioural differences reported in BDD (Malcolm et al., 2021). It may be that a single cognitive profile is unlikely to be representative of the heterogeneous presentation of BDD. The data do, however, indicate that establishing a comprehensive understanding of local/global processing in BDD is important.

An emerging finding is that salience appears influential in BDD. For example, findings in visual and Gestalt perception revealed that impairments are more consistently demonstrated for salient stimuli, such as faces, than for general stimuli. This finding is also demonstrated in the higher-order sub-domains of selective attention and emotion perception, as well as at the executive function level in emotional inhibition, for the processing of appearance-related words. Thus, employing a hierarchical model of cognition reveals that BDD is not simply associated with ‘bottom-up’ visual problems alone. Instead, cognitive differences were most consistently demonstrated in the higher level, ‘top-down’ domains of executive function and social cognition. While less consistent, they were also noted at the lower levels, suggesting the likelihood of an interconnected ‘network of influence’ in which co-occurring cognitive differences interact across the hierarchy. Multi-level modelling with comprehensive cognitive datasets will need to be conducted to further elucidate such a supposition.

This review has highlighted that cognitive difficulties in BDD are present and increase in consistency with increasing cognitive complexity. At present, cognitive deficits are not specifically treated in BDD treatment models, and these data suggest that they should be.

### Limitations

Comparison of findings within sub-domains was difficult due to the great variation in methodology used across the studies, and some inconsistencies in the findings can be accounted for by these variations. Differences in tasks and task characteristics, including in stimuli, presentation time and sequencing, have been noted to account for some of these discrepancies. There is also some overlap across cognitive domains, as tasks generally tap into multiple cognitive processes, which may blur domain-specific differences. However, there is also evidence of individual differences in how neurocognition in BDD is experienced (e.g. sub-groups), although this heterogeneity is unsurprising given the diversity and complexity in the disorder.

The aim of this study was to conduct a systematic review. It was however noted that the small number of studies per sub-domain and variations in task methodologies prevented a meta-analytic synthesis. Furthermore, we limited the review to examining ‘cold’ neurocognition, and not broader cognition, including psychological, emotional and evaluative elements. The influence of psychological ‘hot’ cognition, such as underlying emotions, schemas, beauty ideals and beliefs regarding the importance of attractiveness, also factor in individuals’ perception of their ugliness; however, this is beyond the scope of this review. Yet, our findings regarding the importance of salience in potentially contributing to neurocognitive differences in BDD suggest ‘hot cognition’ is likely to be part of the total cognitive picture of BDD. The cross-sectional designs of the included studies also limited the ability to examine the relationship between neurocognitive profiles and the disorder, including whether these profiles represent a risk factor for its development and maintenance or are a consequence of the disorder itself. Longitudinal investigation is needed to examine whether neurocognitive differences in BDD can be impacted by treatment.

Compared to other conditions, the field of research focused on cognition in BDD is still nascent, and the current literature likely under-represents the diversity of neurocognitive experience among people with the disorder. Thus, it is unclear if the findings are generally representative of the condition, or whether they may relate more to certain sub-groups. Furthermore, while the studies were of acceptable quality with low risk of bias, most studies employed Western and predominantly female samples. Future research should investigate potential neurocognitive differences in samples of diverse cultural backgrounds and genders.

### Future directions

Further investigation of neurocognitive differences in BDD employing a standardised, valid assessment battery longitudinally across larger samples is warranted. The review highlighted neurocognitive abilities that are yet to be examined in BDD. Sub-domains such as interoception, for example, have been proposed to play an important role in unconscious perception errors, which may underlie BDD (Jenkinson and Rossell, 2024). Future reviews could examine patterns of neurocognitive differences between BDD and related disorders such as obsessive-compulsive disorder and anorexia nervosa to better characterise patterns specific to BDD.

## Conclusion

This review contributes to understanding the complex experiences of BDD from a ‘cold’ cognition perspective utilising a neurocognitive hierarchy framework. The literature proffers evidence of behavioural differences in neurocognitive tasks across all levels of the neurocognitive hierarchy, particularly for emotionally salient stimuli and local/global processing biases. Inconsistencies in the findings may be due to methodological variations, or heterogeneity in how individuals experience the disorder. Research employing a comprehensive, standardised test battery of neurocognitive tasks across the hierarchy utilising large samples is needed to further define the neurocognitive profile of BDD.

## Supplemental Material

sj-docx-1-anp-10.1177_00048674241309747 – Supplemental material for A systematic review of neurocognition and social cognition in body dysmorphic disorder Supplemental material, sj-docx-1-anp-10.1177_00048674241309747 for A systematic review of neurocognition and social cognition in body dysmorphic disorder by Katrina Holmes à Court, Amy Malcolm, Wei Lin Toh and Susan L Rossell in Australian & New Zealand Journal of Psychiatry

sj-docx-2-anp-10.1177_00048674241309747 – Supplemental material for A systematic review of neurocognition and social cognition in body dysmorphic disorder Supplemental material, sj-docx-2-anp-10.1177_00048674241309747 for A systematic review of neurocognition and social cognition in body dysmorphic disorder by Katrina Holmes à Court, Amy Malcolm, Wei Lin Toh and Susan L Rossell in Australian & New Zealand Journal of Psychiatry
