# Supplementary material for: A systematic review of neurocognition and social cognition in body dysmorphic disorder
Source: Aust N Z J Psychiatry. 2025 Jan 7;59(3):224–47. doi: 10.1177/00048674241309747 (PMC11837421; doi:10.1177/00048674241309747)
Supplement: sj-docx-1-anp-10.1177_00048674241309747 – Supplemental material for A systematic review of neurocognition and social cognition in body dysmorphic disorder [file sj-docx-1-anp-10.1177_00048674241309747.docx]

**Supplementary Material A**

**Search Strategy**

The search was conducted in the electronic databases of OVID Medline, Scopus, PsycNET and Web of Science (from each database's inception date to June 2024). A secondary search was then conducted in Google Scholar to retrieve grey literature. The search syntax was tailored for each database focusing on keywords in titles and abstracts. Study designs involving both clinically diagnosed BDD participants and healthy controls were selected. Searches were limited to English language, and peer-reviewed sources. Search strings for each database are shown below.

**Search Results**

Total records in Endnote = 9298, after removal of duplicates = 8411 (887 duplicates)

Grey literature search = 3

Records screened = 8414

Articles assessed for eligibility = 146

Articles excluded = 92

Articles included = 54

| Date | Database | Search Terms | Limits | Records |
| --- | --- | --- | --- | --- |
| 4/3/24 | Scopus | "body dysmorphic disorder" OR "body dysmorphia" OR dysmorphophobia AND cogniti* | No filters/limits | 6132 |
| 4/3/24 | OVID Medline | MESH terms: somatoform disorders/ body dysmorphic disorders/ cognition/ executive function/ learning/ mentalization/ perception/ social perception/ social cognition/ space perception/ taste perception/ time perception/ touch perception/ visual perception/ weight perception/ "theory of mind"/ Processing Speed/ Executive Function/ or Cognition Disorders/ Cognitive Dysfunction/ Memory/px [Psychology], Attention/px [Psychology]  Keywords: dysmorphophobia, BDD, neurocognition, organization, organisation, flexibility, fluency, empathy, attention, processing | No filters/limits | 79 |
| 4/3/24 | Web of Science | dysmorphophobia OR "body dysmorphia" OR "body dysmorphic disorder" AND "cogniti*” OR “social cogniti*” OR “neurocogniti*” OR “neuropsy*” OR “visual*” OR “percept*” OR “global” OR “local” OR “emotion recognition” OR “recognition” OR “emotion processing” OR “process” OR “theory of mind” OR “empath” OR “attention*” OR “set-shift” OR “vigilance” OR “executive function” OR “decision-making” OR “memory” OR “mnestic” OR “fluency” OR “flexibility” OR “language” OR “verbal” OR non-verbal” OR “motor” OR “inhibition” OR “dysfunction” OR “impair*” OR “face” OR “facial” OR “organization*” OR organizational*" | No filters/limits | 951 |
| 4/3/24 | PsycNet | Any Field: cogniti* OR neurocogniti* OR neuropsy* OR visual* OR percept* OR global OR local OR recognition OR processing OR processed OR “theory of mind” OR empath* OR attention* OR "set-shift” OR vigilance OR “executive function” OR “decision-making” OR memory OR mnestic OR fluency OR flexibility OR language OR verbal OR "non-verbal” OR motor OR inhibition OR dysfunction OR impair* OR face OR facial OR organization* OR organizational* AND Any Field: "body dysmorphic disorder” OR BDD OR “body dysmorphia” OR dysmorphophobia | No filters/limits | 2136 |

**Database Search Strings**

***OVID Medline***

| String Number | Searches | Results |
| --- | --- | --- |
| 1 | somatoform disorder/ or body dysmorphic disorder | 10897 |
| 2 | dysmorphophobia.mp | 205 |
| 3 | dysmorphoph*.mp. [mp=title, book title, abstract, original title, name of substance word, subject heading word, floating sub-heading word, keyword heading word, organism supplementary concept word, unique identifier, synonyms, population supplementary concept word, anatomy supplementary concept word] | 256 |
| 4 | BDD.mp. | 2188 |
| 5 | cognition/ or executive function/ or learning/ or mentalization/ or perception/ or social perception/ or social cognition/ or space perception/ or taste perception/ or time perception/ or touch perception/ or visual perception/ or weight perception/ or “theory of mind”/ | 383631 |
| 6 | 1 or 3 or 4 | 12641 |
| 7 | 5 and 6 | 287 |
| 8 | neurocognition.mp. | 3171 |
| 9 | Processing Speed/ | 129 |
| 10 | Executive Function/ or Cognition Disorders/ | 84481 |
| 11 | Memory/px[Psychology] | 1 |
| 12 | organisation.mp. | 41521 |
| 13 | organization.mp. | 867915 |
| 14 | flexibility.mp. | 101190 |
| 15 | Cognitive Dysfunction/ or fluency.mp. | 53766 |
| 16 | empathy.mp. | 33803 |
| 17 | Attention/px[Psychology] | 11 |
| 18 | emotion.mp. | 59445 |
| 19 | processing.mp. | 717081 |

***PsychNET String***

| Searches | Results |
| --- | --- |
| Body dysmorphic disorder” OR “BDD” OR “body dysmorphia” OR “dysmorphophobial” AND (“”cogniti*” OR “social cogniti*” OR “neurocogniti*” OR “neuropsy*” OR “visual*” OR “percept*” OR “global” OR “local” OR “emotion recognition” OR “recognition” OR “emotion processing” OR “process” OR “theory of mind” OR “empath” OR “attention*” OR “set-shift” OR “vigilance” OR “executive function” OR “decision-making” OR “memory” OR “mnestic” OR “fluency” OR “flexibility” OR “language” OR “verbal” OR “non-verbal” OR “motor” OR “inhibition” OR “dysfunction” OR “impair*” OR “face” OR “facial” OR “organization*” OR “organization*” OR “organizational*” AND APA Full-Text Only AND Peer-Reviewed Journals only | 2136 |

***Web of Science***

| Searches | Results |
| --- | --- |
| ALL=(dysmorphophobia OR “body dysmorphia” OR “body dysmorphic disorder” AND “cogniti*” OR “social cogniti*” OR “neurocogniti*” OR “neuropsy*” OR “visual*” OR “percept*” OR “global” OR “local” OR “emotion recognition” OR “recognition” OR “emotion processing” OR “process” OR “theory of mind” OR “empathy” OR “attention*” OR “set-shift” OR “vigilance” OR “executive function” OR “decision-making” OR “memory” OR “mnesic” OR “fluency” OR “flexibility” OR “language” OR “verbal” OR “non-verbal” OR “motor” OR “inhibiton” OR “dysfunction” OR “impair*” OR “face” OR “facial” OR “organization*” OR organizational*”) | 16, 128, 873 |

***Scopus***

| Searches | Results |
| --- | --- |
| “body dysmorphic disorder” OR “body dysmorphia” dysmorphophobia AND “cogniti* | 6132 |
