# Supplementary material for: A systematic review of neurocognition and social cognition in body dysmorphic disorder
Source: Aust N Z J Psychiatry. 2025 Jan 7;59(3):224–47. doi: 10.1177/00048674241309747 (PMC11837421; doi:10.1177/00048674241309747)
Supplement: sj-docx-2-anp-10.1177_00048674241309747 – Supplemental material for A systematic review of neurocognition and social cognition in body dysmorphic disorder [file sj-docx-2-anp-10.1177_00048674241309747.docx]

**Supplementary Material B**

**Table 4**

*Newcastle-Ottawa Scale Quality Assessment of Included Studies*

| **Study** | **Selection of case and controls** | | | | | **Comparability of cases and controls** | | | **Exposure** | | | | | | |  |
| --- | --- | --- | --- | --- | --- | --- | --- | --- | --- | --- | --- | --- | --- | --- | --- | --- |
|  | BDD definition | BDD select | HC select | HC definition | | | Design comparability | | | Exposure | | BDD:HC | | | Completion | |
| Buhlmann et al. (2006) | SCID; BDD-YBOCS ⋆ | BDD patients, Massachusetts General Hospital ⋆ | Community ⋆ | | No mention of history of outcome (no psychiatric history) | | | Different education, non-significant difference age, sex * | | | Medical records; SCID; BDD-YBOCS ⋆ | | Yes ⋆ | No non-responses across groups ⋆ | |  |
| Buhlmann et al. (2011a) | SCID, BDD-YBOCS ⋆ | Community ⋆ | Community ⋆ | | No psychiatric history; SCID ⋆ | | | No significant difference in age, sex, education ** | | | SCID ⋆ | | Yes ⋆ | No non-responses across groups ⋆ | |  |
| Buhlmann et al. (2004) | SCID; BDD-YBOCS ⋆ | BDD patients, Massachusetts General Hospital ⋆ | Community ⋆ | | No mention of history of outcome (no psychiatric history) | | | Yoked design ⋆⋆ | | | Medical records; SCID; BDD-YBOCS ⋆ | | Yes ⋆ | No non-responses across groups ⋆ | |  |
| Buhlmann et al. (2002) | DSM-IV BDD SCID ⋆ | BDD patients, Massachusetts General Hospital ⋆ | Community ⋆ | | No psychiatric history; clinical interview SCID-I/P ⋆ | | | No significant difference in age, education * | | | Medical records; SCID; BDD-YBOCS ⋆ | | Yes ⋆ | No non-responses across groups ⋆ | |  |
| Buhlmann et al. (2014) | SCID, BDD-YBOCS ⋆ | Community ⋆ | Community ⋆ | | No psychiatric history; SCID ⋆ | | | No significant difference in age, sex * | | | SCID ⋆ | | Yes ⋆ | No non-responses across groups ⋆ | |  |
| Buhlmann et al. (2011b) | SCID, BDD-YBOCS ⋆ | Community ⋆ | Community ⋆ | | No psychiatric history; SCID ⋆ | | | No significant difference in age, sex * | | | SCID ⋆ | | Yes ⋆ | No non-responses across groups ⋆ | |  |
| Buhlmann et al. (2015a) | SCID, BDD-YBOCS ⋆ | Community ⋆ | Community ⋆ | | No psychiatric history; SCID ⋆ | | | No significant difference in age, sex, education ** | | | SCID ⋆ | | Yes ⋆ | No non-responses across groups ⋆ | |  |
| Buhlmann et al. (2013) | SCID, BDD-YBOCS ⋆ | Community ⋆ | Community ⋆ | | No psychiatric history; SCID ⋆ | | | No significant difference in age, sex, education ** | | | SCID ⋆ | | Yes ⋆ | No non-responses across groups ⋆ | |  |
| Chen et al. (2023) | SCID, BDD-DSM-5 ⋆ | Karolinska Institute | Community ⋆ | | No psychiatric history; SCID, MINI ⋆ | | | No significant difference in age, sex, education ⋆⋆ | | | SCID ⋆ | | Yes ⋆ | No non-responses across groups ⋆ | |  |
| Deckersbach et al. (2000) | SCID; BDD-YBOCS ⋆ | Hospital outpatients, Butler Hospital BDD Program ⋆ | Community ⋆ | | No psychiatric history; SCID ⋆ | | | No significant difference in age, sex, education, verbal intelligence, handedness ⋆⋆ | | | SCID ⋆ | | Yes ⋆ | No non-responses across groups ⋆ | |  |
| Dunai et al. (2010) | SCID, BDDM; out-patients | BDD outpatients; psychiatrist records | Community ⋆ | | No psychiatric history; SCID ⋆ | | | No significant difference in age, sex, education ⋆⋆ | | | SCID; BDD-YBOCS ⋆ | | Yes ⋆ | No non-responses across groups ⋆ | |  |
| Fang et al. (2020) | DSM-IV-TR; SCID; BDD-YBOCS ⋆ | BDD outpatients⋆ | Community ⋆ | | No psychiatric history; SCID ⋆ | | | No significant difference in age ⋆ | | | SCID; BDD-YBOCS ⋆ | | Yes ⋆ | No non-responses across groups ⋆ | |  |
| Feusner et al. (2010a) | DSM-IV; BDDM; BDD-YBOCS ⋆ | BDD outpatients ⋆ | Community ⋆ | | No psychiatric history; clinical interview; MINI ⋆ | | | No significant difference in age, sex, education ⋆⋆ | | | BDDM; BDD-YBOCS, MINI ⋆ | | Yes ⋆ | No non-responses across groups ⋆ | |  |
| Feusner et al. (2011) | SCID; BDDM; BDD-YBOCS ⋆ | BDD patients ⋆ | Community ⋆ | | No current /past Axis I disorder; SCID; MINI ⋆ | | | No significant difference in age, sex, education ⋆⋆ | | | BDDM; BDD-YBOCS, MINI ⋆ | | Yes ⋆ | No non-responses across groups ⋆ | |  |
| Feusner et al. (2010b) | SCID; BDDM; BDD-YBOCS ⋆ | BDD patients ⋆ | Community ⋆ | | No psychiatric history; clinical interview; MINI ⋆ | | | No significant difference in gender, age, handedness, education ⋆⋆ | | | BDDM; BDD-YBOCS, MINI ⋆ | | Yes ⋆ | No non-responses across groups ⋆ | |  |
| Feusner et al. (2007) | DSM-IV; BDDM; BDD-YBOCS ⋆ | BDD outpatients ⋆ | Community ⋆ | | No psychiatric history; SCID ⋆ | | | No significant difference in age, sex, education ⋆⋆ | | | SCID; BDDM, MINI ⋆ | | Yes ⋆ | No non-responses across groups ⋆ | |  |
| Giannopolous et al. (2022) | DSM-IV BDD; BDD-YBOCS; DCQ ⋆ | Community ⋆ | Community ⋆ | | No psychiatric history; clinical interview; ⋆ | | | No significant difference in age, sex ⋆ | | | DSM-IV BDD; BDD-YBOCS; DCQ ⋆ | | Yes ⋆ | No non-responses across groups ⋆ | |  |
| Grace et al. (2019) | SCID; BDDM; BDD-YBOCS ⋆ | BDD patientsI ⋆ | Community ⋆ | | No psychiatric history; clinical interview; MINI ⋆ | | | No significant difference in sex, age, and years of education ⋆⋆ | | | BDDM; BDD-YBOCS, MINI ⋆ | | Yes ⋆ | No non-responses across groups ⋆ | |  |
| Greenberg et al. (2014) | SCID, BDD-YBOCS; ⋆ | BDD outpatients ⋆ | Community ⋆ | | No psychiatric history; SCID ⋆ | | | No significant difference in age, sex ⋆ | | | SCID; BDD-YBOCS ⋆ | | Yes ⋆ | Unequal, BDD (n = 1) | |  |
| Greenberg et al. (2018) | SCID, BDD-YBOCS; ⋆ | BDD outpatients ⋆ | Community ⋆ | | No psychiatric history; SCID ⋆ | | | No significant difference in age, sex ⋆ | | | SCID; BDD-YBOCS ⋆ | | Yes ⋆ | Unequal, BDD (n = 4) | |  |
| Grocholeswki et al. (2012) | SCID; BDDM; BDD-YBOCS ⋆ | BDD outpatientsI ⋆ | Community controls ⋆ | | No psychiatric history; clinical interview; MINI ⋆ | | | No significant difference in sex, age ⋆ | | | BDDM; BDD-YBOCS, MINI ⋆ | | Yes ⋆ | No non-responses across groups ⋆ | |  |
| Hanes (1998) | SCID; patients ⋆ | Clinic patients; psychiatrist records ⋆ | Unspecified | | Unspecified | | | Unmatched confounder variables | | | Unspecified | | Unspecified | No non-responses across groups ⋆ | |  |
| Hartmann et al. (2015) | SCID, BDD-YBOCS ⋆ | Hospital patients ⋆ | Community ⋆ | | No psychiatric history; clinical interview ⋆ | | | No significant difference in sex, age, race, ethnicity, marital status ⋆⋆ | | | SCID; BDD-YBOCS ⋆ | | Yes ⋆ | HC (equal ⋆) | |  |
|  |  |  |  | |  | | |  | | |  | |  |  | |  |
| Hübner et al. (2016) | SCID; BDD-YBOCS ⋆ | Community ⋆ | Community ⋆ | | No psychiatric history; clinical interview ⋆ | | | No significant difference in sex, age, education ⋆⋆ | | | SCID; BDD-YBOCS ⋆ | | Yes ⋆ | No non-responses across groups ⋆ | |  |
| Jefferies et al. (2012) | SCID, BDD-YBOCS; ⋆ | BDD clinic outpatients ⋆ | Community ⋆ | | No psychiatric history; SCID ⋆ | | | No significant difference in age, IQ, education ⋆⋆ | | | SCID; BDD-YBOCS ⋆ | | Yes ⋆ | No non-responses across groups ⋆ | |  |
| Jefferies-Sewell et al. (2017) | SCID, BDD-YBOCS, ⋆ | Outpatients ⋆ | Undergraduates | | BDD-YBOCS (< 10) | | | Matched age, education, IQ ⋆⋆ | | | BDD-YBOCS ⋆ | | Yes ⋆ | No non-responses across groups ⋆ | |  |
| Kaplan et al. (2014) | SCID; BDDM; DCQ ⋆ | St Vincent’s clinic patients ⋆ | Community ⋆ | | No psychiatric history, MINI ⋆ | | | No significant difference in age, sex, IQ ⋆⋆ | | | SCID; BDD-YBOCS; DCQ ⋆ | | Yes ⋆ | No non-responses across groups ⋆ | |  |
| Kerwin et al. (2014) | SCID; BDDM; BDD-YBOCS ⋆ | Community ⋆ | Community ⋆ | | No psychiatric history, MINI ⋆ | | | Equivalent age, education, sex ⋆⋆ | | | SCID; BDDM, MINI ⋆ | | Yes ⋆ | No non-responses across groups ⋆ | |  |
| Kollei et al. (2017) | SCID; BDDM; BDD-YBOCS ⋆ | Community ⋆ | Community ⋆ | | No psychiatric history, MINI ⋆ | | | No significant difference in sex (all f), age, education ⋆⋆ | | | SCID; MINI, ; BDD-YBOCS ⋆ | | Yes ⋆ | No non-responses across groups ⋆ | |  |
| Lambrou et al. (2011) | DSM-IV; BDD-YBOCS ⋆ | Priory Hospital; support groups ⋆ | Community ⋆ | | HC no psychiatric history; arts/design HC with degrees ⋆ | | | No significant difference in sex, age ⋆ | | | DSM-IV; BDD-YBOCS ⋆ | | Yes ⋆ | No non-responses across groups ⋆ | |  |
| Li et al. (2015) | DSM-IV BDD; SCID; BDDM; BDD-YBOCS ⋆ | Clinic patients ⋆ | Community ⋆ | | No psychiatric history, MINI ⋆ | | | No significant difference in sex, age, education ⋆⋆ | | | BDDM; BDD-YBOCS; MINI ⋆ | | Yes ⋆ | No non-responses across groups ⋆ | |  |
| Malcolm et al. (2021) | DSM-IV BDD; MINI; BDDM; BDD-YBOCS | Clinic BDD patients, community ⋆ | Community ⋆ | | No psychiatric history, MINI | | | No significant difference in sex, age, and IQ ⋆⋆ | | | BDDM; BDD-YBOCS; MINI ⋆ | | Yes ⋆ | No non-responses across groups ⋆ | |  |
| Monzani et al. (2013) | DSM-IV BDD; SCID, BDDM; BDD-YBOCS ⋆ | Maudsley Hospital outpatients; BDD support groups ⋆ | Undergraduates; community ⋆ | | No psychiatric history Axis I ⋆ | | | Matched age, sex, education ⋆⋆ | | | BDDM; BDD-YBOCS; MINI ⋆ | | Yes ⋆ | No non-responses across groups ⋆ | |  |
| Moody et al. (2015) | DSM-IV BDD, MINI, BDDM; BDD-YBOCS ⋆ | Community ⋆ | Community ⋆ | | No psychiatric history, MINI ⋆ | | | Matched for sex, age, education ⋆⋆ | | | BDDM; BDD-YBOCS; MINI ⋆ | | Yes ⋆ | No non-responses across groups ⋆ | |  |
| Onken et al. (2024) | DSM-IV BDD; SCID; BDD-YBOCS ⋆ | Outpatients, community ⋆ | Community ⋆ | | No psychiatric history, SCID ⋆ | | | Matched for sex (all f), age, education ⋆⋆ | | | SCID; BDD-YBOCS ⋆ | | Yes ⋆ | No non-responses across groups ⋆ | |  |
| Pikoos et al. (2024) | DSM-IV BDD, MINI, BDDM; BDD-YBOCS ⋆ | Outpatients, community ⋆ | Community ⋆ | | No psychiatric history, MINI ⋆ | | | No significant difference in sex, age, and IQ ⋆⋆ | | | BDDM; BDD-YBOCS; MINI ⋆ | | Yes ⋆ | No non-responses across groups ⋆ | |  |
| Reese et al. (2010) | DSM-IV BDD, SCID; BDDM; BDD-YBOCS ⋆ | Clinic BDD patients ⋆ | Community ⋆ | | No psychiatric history ⋆ | | | Matched age, education ⋆ | | | Clinic records, DSM-IV; BDDM; SCID ⋆ | | Yes ⋆ | No non-responses across groups ⋆ | |  |
| Reese et al. (2011a) | Clinical interview; records; BDDQ; BDDM ⋆ | Hospital outpatients ⋆ | Community ⋆ | | Clinical interview; no psychiatric history ⋆ | | | No significant difference in education, age, and IQ ⋆⋆ | | | DSM-IV; BDDM; SCID; BDD-YBOCS; BABS ⋆ | | Yes ⋆ | No non-responses across groups ⋆ | |  |
| Reese et al. (2011b) | DSM-IV BDD; SCID; BDD-YBOCS ⋆ | Hospital outpatients ⋆ | Community ⋆ | | Clinical interview; No current/past Axis I except phobia/AA ⋆ | | | No significant difference in education, age, and IQ ⋆⋆ | | | SCID; BDDM; BDD-YBOCS; BABS ⋆ | | Yes ⋆ | No non-responses across groups (HC) ⋆ | |  |
| Ritter et al. (2020) | DSM-IV BDD, SCID; BDDM; BDD-YBOCS ⋆ | BDD clinic outpatients, Goethe University, community ⋆ | Community controls ⋆ | | Clinical interview, SCID; no current/past Axis I disorder ⋆ | | | Age and gender matched ⋆⋆ | | | Clinical records, clinical interview, diagnosis; SCID; BDDM; BDD-YBOCS ⋆ | | Yes ⋆ | No non-responses across groups ⋆ | |  |
| Rossell et al. (2014) | DSM-IV, BDD; SCID; BDDM; DCQ; BDD-YBOCS ⋆ | BDD clinic patients, community ⋆ | Community controls ⋆ | | Clinical interview; SCID; no psychiatric history ⋆ | | | Age, gender, education matched ⋆⋆ | | | Clinical records; SCID; BDDM; BDD-YBOCS; DCQ ⋆ | | Yes ⋆ | No non-responses across groups ⋆ | |  |
| Silverstein et al. (2015) | DSM-IV BDD; SCID; BDD-YBOCS ⋆ | Hospital outpatients ⋆ | Community ⋆ | | Clinical interview; no psychiatric history ⋆ | | | No significant difference in sex only ⋆ | | | Clinical records; SCID; BDD-YBOCS ⋆ | | Yes ⋆ | No non-responses across groups ⋆ | |  |
| Stangier et al. (2008) | DSM-IV BDD; SCID; BDDM; BDD-YBOCS; DCQ ⋆ | Dermatology clinic patients ⋆ | Community controls ⋆ | | Clinical interview; SCID; BDDM; no psychiatric history ⋆ | | | No significant difference in education, age ⋆⋆ | | | Dermatologist ratings; SCID; BDDM; BDD-YBOCS; DCQ ⋆ | | Yes ⋆ | No non-responses across groups ⋆ | |  |
| Toh et al. (2015a) | MINI; BDDM; BDD-YBOCS ⋆ | BDD outpatients, community ⋆ | Community, volunteer database ⋆ | | Clinical interview; no psychiatric history (self, immediate family) ⋆ | | | No significant difference in sex, age, and IQ ⋆⋆ | | | Clinical records; MINI; BDD-YBOCS ⋆ | | Yes ⋆ | No non-responses across groups ⋆ | |  |
| Toh et al. (2015b) | MINI; BDDM; BDD-YBOCS ⋆ | BDD outpatients, community ⋆ | Community, volunteer database ⋆ | | Clinical interview; no psychiatric history (self, immediate family) ⋆ | | | No significant difference in sex, age, and IQ ⋆⋆ | | | Clinical records; MINI; BDD-YBOCS ⋆ | | Yes ⋆ | No non-responses across groups ⋆ | |  |
| Toh et al. (2017a) | MINI; BDDM; BDD-YBOCS ⋆ | BDD outpatients, community ⋆ | Community, volunteer database ⋆ | | Clinical interview; no psychiatric history (self, immediate family) ⋆ | | | Matched age, sex, IQ; BDD < HC education ⋆⋆ | | | Clinical records; MINI; BDD-YBOCS ⋆ | | Yes ⋆ | No non-responses across groups ⋆ | |  |
| Toh et al. (2017b) | MINI; BDDM; BDD-YBOCS ⋆ | BDD outpatients, community ⋆ | Community, volunteer database ⋆ | | Clinical interview; no psychiatric history (self, immediate family) ⋆ | | | Matched age, sex, IQ; BDD < HC education ⋆⋆ | | | Clinical records; MINI; BDD-YBOCS ⋆ | | Yes ⋆ | No non-responses across groups ⋆ | |  |
| Toh et al. (2017c) | MINI; BDDM; BDD-YBOCS ⋆ | BDD outpatients, community ⋆ | Community, volunteer database ⋆ | | Clinical interview; no psychiatric history (self, immediate family) ⋆ | | | Matched age, sex, IQ; BDD < HC education ⋆⋆ | | | Clinical records; MINI; BDD-YBOCS ⋆ | | Yes ⋆ | No non-responses across groups ⋆ | |  |
| Waldorf et al. (2019) | DSM-5 BDD; MDSQ; SCID ⋆ | Community ⋆ | Community ⋆ | | No current suicidality, schizophrenia, bipolar, substance use disorder; SCID ⋆ | | | Matched for sex (all m), age, education ⋆⋆ | | | DSM-IV BDD; MDSQ; SCID ⋆ | | Yes ⋆ | No non-responses across groups ⋆ | |  |
| Wang et al. (2024) | DSM-IV BDD, SCID; BDD-YBOCS ⋆ | BDD outpatients ⋆ | Undergraduates ⋆ | | Clinical interview; no psychiatric history ⋆ | | | No significant difference in age ⋆ | | | DSM-IV BDD, SCID; BDD-YBOCS ⋆ | | Yes ⋆ | No non-responses across groups ⋆ | |  |
| Wilhelm et al. (2003) | DSM-IV BDD, SCID; BDD-YBOCS ⋆ | BDD outpatients ⋆ | Community ⋆ | | Clinical interview; no psychiatric history ⋆ | | | Matched age, education, IQ ⋆⋆ | | | Clinical records; SCID; BDD-YBOCS ⋆ | | Yes ⋆ | No non-responses across groups ⋆ | |  |
| Wong et al. (2021) | MINI, BDDM; BDD-YBOCS ⋆ | Community ⋆ | Community ⋆ | | Clinical interview; MINI, no psychoactive medications for 8 weeks ⋆ | | | No significant difference in sex, age ⋆⋆ | | | BDDM; BDD-YBOCS; MINI ⋆ | | Yes ⋆ | No non-responses across groups ⋆ | |  |
| Yaryura-Tobias et al. (2002) | DSM-IV BDD, SCID; BDD-YBOCS ⋆ | BDD patients ⋆ | Community ⋆ | | Clinical interview; no psychiatric history ⋆ | | | Unmatched age, sex, education | | | Clinical records; SCID; BDD-YBOCS ⋆ | | Yes ⋆ | No non-responses across groups ⋆ | |  |
| Yousefi et al. (2020) | DSM-IV BDD, SCID; BDD-YBOCS ⋆ | BDD outpatients ⋆ | Community ⋆ | | Clinical interview, SCID; no current Axis I disorder, no psychoactive medication ⋆ | | | Matched age, sex, education ⋆⋆ | | | Clinical records, clinical interview, SCID, diagnosis ⋆ | | Yes ⋆ | No non-responses across groups ⋆ | |  |

*Note:* Abbreviations: Body Dysmorphic Disorder (BDD), Body Dysmorphic Disorder-Diagnostic Module (BDD-DM), Dysmorphic Concern Questionnaire (DCQ), Healthy Controls (HC), MINI International Neuropsychiatric Interview (MINI), Muscle Dysmorphia Symptom Questionnaire (MDSQ), Yale-Brown Obsessive-Compulsive Scale modified for Body Dysmorphic Disorder (BDD-YBOCS), *Diagnostic and Statistical Manual of Mental Disorders* (4th ed. Body Dysmorphic Disorder Module; DSM-IV BDD), *Diagnostic and Statistical Manual of Mental Disorders* (5th ed. Body Dysmorphic Disorder Module; *DSM–5* BDD), Structured Clinical Interview for DSM-IV (SCID).
